# Identification of Annonaceous Acetogenins and Alkaloids from the Leaves, Pulp, and Seeds of *Annona atemoya*

**DOI:** 10.3390/ijms24032294

**Published:** 2023-01-24

**Authors:** Bassam S. M. Al Kazman, Joanna Elizabeth Harnett, Jane Rouse Hanrahan

**Affiliations:** The School of Pharmacy, Faculty of Medicine and Health, The University of Sydney, Camperdown, NSW 2006, Australia

**Keywords:** *Annona atemoya*, leaves, fruit’s pulp, seeds, annonaceous acetogenins, alkaloids

## Abstract

Annonaceae is a large family composed of more than 119 genera and more than 2500 species that are distributed in both tropical and subtropical areas. The *Annona* genus is a member of Annonaceae family, which encompasses about 175 species, most of which are native to Brazil and tropical America. This plant is commonly found on tropical and subtropical continents. *Annona atemoya* is a commercially important hybrid of *A. squamosa* and *A. cherimola*. Phytochemical investigations of *A. atemoya* leaves, fruit, and seeds have been conducted in limited studies. The purpose of this study was to investigate the constituents of the leaves, fruit pulp, and seeds of *A. atemoya* because few studies have reported their constituents. Annonaceous acetogenins were identified in the leaves and pulp of *A. atemoya* for the first time. Twenty compounds were identified: sixteen were acetogenins and four were alkaloids. Additionally, two compounds were isolated, and their structures were confirmed by spectroscopic analysis and compared with the results of previous studies. The concentration of acetogenins in the pulp was very low compared with that in the leaves, whereas the seeds were found to contain the highest concentrations and greatest diversity of compounds.

## 1. Introduction

Annonaceae is a large plant family composed of more than 119 genera and more than 2500 species that are distributed across tropical and subtropical areas. This family is chemically characterised by the existence of various phytochemical constituents such as alkaloids, annonaceous acetogenins (ACs), volatile oils, and phenolic compounds [1]. The *Annona* genus is a member of the Annonaceae family that encompasses approximately 175 species, many of them native of Brazil and tropical America [1,2,3]. Due to their edible fruits and medicinal proprieties, *Annona* is the most important genus of the Annonaceae family [1]. *Annona atemoya* (*A. atemoya*) belongs to the family of Annonaceae known as custard apple [1,2,3]. This plant is commonly found in tropical and subtropical areas such as Asia, Africa, South and North America, and Australia [4]. *A. atemoya* is a hybrid of two *Annona* species: *Annona squamosa* (sugar apple) and *Annona cherimola* (cherimoya), first reported in 1907 [3,4]. Various cultivars have been reported for *A. atemoya,* and the most common ones grown in Australia are African Pride, KJ Pink’s Mammoth, Island Gem, Nielsen, Hillary White, and Maroochy Gold [4]. Despite the fact that there are many *Annona* species, only a limited number of this family are economically important, and *A. atemoya* is one of them. In Australia, this plant is commonly known as the “custard apple”, having a sweet and creamy, custard-like fruit. It is increasingly available in most retail food stores [3]. There is also some anecdotal evidence reported by growers of the leaves being purchased for making teas similar to those made with *atemoya* species and used in traditional medicines.

Many *Annona* species have a long history of use in traditional medicines, and the constituents have been well studied and are known to exhibit a range of biological activities [5,6,7,8]. However, the phytochemical investigations of the leaves, fruits, and seeds of the newer hybrid *A. atemoya* have been reported only in a limited number of studies. An initial study reported the isolation of alkaloids such as lanuginosine, liriodenine, lysicamine, pronuciferine, anonaine, asimilobine, and stepharine from the leaves [1]. A more recent study reported the isolation of additional alkaloids such as dehydroanomuricine-*N*-oxide, anomuricine, reticuline, scoulerine, nornuciferine, and norisocorydine [9]. The leaves of *A. atemoya* were found to also contain a number of phenolic compounds including quercetin-3-O-rutinoside-7-O-glucoside, quercetin-3-O-rutinoside, quercetin-3-O-glucoside, luteolin-3-galactoside-7-rhamnoside, apigenin-8-C-glucoside, quercetin-3-O-rutinoside-7-O-pentoside, kaempferol-3-galactoside-7-rhamnoside, luteolin-3-glucoside-7-rhamnoside, catechin, and epicatechin [10]. The fruits of *A. atemoya* have been reported to contain a wide range of essential oils, with the main compounds identified being α-pinene, β-pinene, germacrene D, and (*E*)-ocimene [11,12,13]. In addition, the fruit of *A. atemoya* was reported to contain phenolic compounds such as epicatechin, catechin, 3,4-dihydroxybenzoic acid, chlorogenic acid, and *p*-coumaric acid [14]. ACs have been reported as the main phytochemical constituents of the seeds in comparison with alkaloids, *N*-acyl-tryptamines, and phenolic components [15,16,17,18,19,20,21,22]. Approximately 36 ACs have been isolated and identified from the seeds of *A. atemoya* [15,16,17,18,19,20,21,22], 2 alkaloids including atemoine and cleistopholine [23], and 4 phenolic compounds including chlorogenic acid, ferulic acid, vanillic acid, and myricetin [24].

Despite the potential health benefits of *Annona* fruits, high levels of consumption of the fruit, nectar, and/or herbal teas *of A. muricata* L. (Graviola, soursop) have been associated with a higher incidence of atypical parkinsonism or progressive supranuclear palsy (PSP) [25,26,27]. This disease has been correlated to the contents of alkaloids and acetogenins present in *A. muricata*, particularly ACs such as annonacin [28,29]. Annonacin was also found to be toxic to dopaminergic and other mesencephalic cells [30] and to induce the accumulation of tau proteins in the cultured neurons [31]. Given the increasing consumption of custard apples and the few studies reporting their phytochemical constituents, the aim of this study was to further investigate the phytochemical constituents of the leaves, fruit pulp, and seeds of *A. atemoya*.

## 2. Results and Discussion

### 2.1. Thin-Layer Chromatography (TLC) and Column Chromatography (CC)

African Pride (AP) and KJ Pinks Mammoth (KJ) are cultivars of *A. atemoya*, which display similarities in their phytochemical components according to TLC analysis. Therefore, the AP cultivar was studied in detail, whereas the KJ cultivar was only studied via mass spectrometry (MS) (Supplementary Materials Figure S1). Based on the TLC results, all AP parts (leaves, pulp, and seeds) showed the presence of ACs as pink bands after reacting with Kedde’s reagents. The concentration of low polarity ACs (*Rf* 0.65) in the hexane extract of the leaves was very low, shown as a very faint spot. The ethyl acetate extract was the richest in ACs, shown as approximately three separate bands with an *Rf* of between 0.25 and 0.40. The TLC of the pulp suggested that the concentration of ACs was very low, with the ethyl acetate extract displaying two faint spots with *Rf* values of 0.25 and 0.85. Similarly, the hexane extract also exhibited only a very faint spot of a nonpolar AC with an *Rf* of 0.85. To the best of our knowledge, this is the first report of the presence of ACs in the leaves and pulp of *A. atemoya*. All three extracts of the seeds indicated the presence of ACs as pink bands after reacting with Kedde’s reagents (Supplementary Materials Figure S2). Both the hexane and ethanol extracts displayed similar pink bands at an *Rf* of 0.25; however, these spots were significantly less intense compared with those of the ethyl acetate extract. The ethyl acetate extract exhibited the richest diversity of ACs compared with the hexane and ethanol extracts.

Column chromatography under gravity of the crude leaf extract (6.57 g) afforded a total of 163 fractions (10 mL); fractions (110–129; 145.2 mg) were collected in one flask (fraction 1H), as TLC indicated the presence of the lowest polarity ACs in the extract along with other constituents. The TLC of the fractions (130–131; 33.3 mg) indicated the presence of ACs containing some contaminants visible under UV light, and these fractions were combined (fraction 2H). Finally, the TLC of six fractions (138–143; 24 mg) also indicated the presence of very polar ACs, so these fractions were combined (fraction 3H).

Column chromatography of the ethanol extract (12.6 g) eluted with DCM:MeOH (100:0 *v*/*v*) to DCM:MeOH (0:100 *v*/*v*) yielded a total of 276 fractions, which were collected; separation resulted in five fractions, namely: A (240 mg), B (80 mg), C (2.27 g), D (5.9 g), and E (2.60 g). Of the five fractions, only fractions A and B exhibited four orange spots, indicating alkaloids with an *Rf* ranging from 0.42 to 0.85 after visualisation with Dragendorff’s reagent (Supplementary Materials Figure S3). Due to the small amount of the ethyl acetate crude extract, it was directly submitted to flash chromatography for further purification. Both the pulp and seed crude extracts were submitted to NMR and MS analysis due to the small amount of these crude extracts.

### 2.2. Flash Chromatography

The ethyl acetate extract of the leaves was directly purified by flash chromatography due to small amount of the crude extract (2.991 g). Gradient elution of DCM (100:0 *v*/*v*) to DCM:MeOH to MeOH0: 100 *v*/*v* yielded 158 fractions (25 mL), which were analysed by TLC. Similar fractions were combined as follows: fraction 1E (46–66) (121.4 mg) and fraction 2E (67–79) (66.7 mg) containing the lowest polarity ACs; fraction 3E (80–100) (119.4 mg), fraction 4E (101–132) (164.2 mg) and fraction 5E (133–144) (190.9 mg) (Supplementary Materials Figure S4). The further purification by flash chromatography of fractions 1E to 5E using similar gradient methodology is summarised in the Supplementary Materials (Table S1). After the final chromatographic separation, eight fractions were identified as containing ACs.

### 2.3. Nuclear Magnetic Resonance (NMR), Mass Spectrometry (MS), and Tandem Mass Spectrometry (MS/MS)

#### 2.3.1. Leaves

The concentration of ACs in the hexane extract was extremely low compared with that in the ethyl acetate extract, and the four compounds identified were common to the other extracts according to the NMR and MS results. Both NMR and MS indicated the presence of the same ACs in the hexane and ethyl acetate extracts, and the TLC also confirmed a very weak pink spot. The MS fragmentation pathway used to identify ACs has previously been reported [10,32]. The molecular mass was determined as 530 by ESI-MS ([M+H]^+^ *m*/*z* = 531) suggesting a molecular formula C_35_H_62_O_3_. The MSMS indicated the product ion of [M + Li]^+^ (*m*/*z* = 537) with intense fragment peaks at *m*/*z* 313, 301, 285, and 271, allowing localisation of the epoxy ring at C-15/C-16, with the double bond location at C-19/C-20. In the ^1^H-NMR spectrum, the peak at 6.98 ppm was indicative of the deshielded ethylenic proton, and the presence of resonances at δ 2.90 and 2.93 were suggestive of an epoxy ring indicated epoxymurin-A (**1**). The concentration of this compound was lower in ethyl acetate extract than the hexane concentration. Both the NMR and MS results are consistent with two studies that have reported this compound in the pulp and stem bark of *A. muricata* [33,34].

Two epoxy ACs were identified as a mixture of isomeric compounds in both the hexane and ethyl acetate extracts as diepomuricanin A and B (**2** and **3**). The ^1^H-NMR spectrum showed a peak at 6.96 ppm and a molecular mass of 564 *m/z,* corresponding to the molecular formula C_35_H_62_O_4_. The MSMS indicated the product ion of [M + Li]^+^ (*m*/*z* = 553), and the location of the epoxy rings in the alkyl chain for diepomuricanin A at the C-15/C-16 and C-19/C-20 was indicated by the presence of two pairs of peaks at *m*/*z* 383 and 371 and *m*/*z* 313 and 301. For diepomuricanin B, epoxy rings were observed at C-13/C-14 and C-17/C- 18 by the presence of two pairs of peaks at *m*/*z* 355 and 343 and *m*/*z* 285 and 273. These compounds were identified via a comparison of the spectral data with the only study reporting these compounds from the hexane fraction of the methanolic extract of *A. muricata* seeds [35].

Annotemoyin-1 and -2 (**4** and **5**) were identified in the hexane and ethyl acetate extracts, showing peaks in the ^1^H-NMR at 6.98 ppm and a molecular mass of 564 *m*/*z*. The MSMS analysis revealed intense fragments at *m*/*z* 277, 247, and 205, suggesting the localisation of a mono-THF ring at C-18/C-21. These mono tetrahydrofuran ACs were previously reported in the seeds of *A. atemoya* (African Pride cultivar) [21]. An additional four compounds were identified in the ethyl acetate extract; two of them were isolated and structurally characterised. The isolation and identification of ACs from *A. atemoya* leaf has not been previously reported.

The structures of the pure compounds were elucidated based on NMR experiments and are consistent with those previously reported for these compounds containing two adjacent bis-tetrahydrofuran rings: squamocin G (**6**) (11.2 mg) and squamocin C (**7**) (9 mg) [36,37,38]. Compound **6** (C_37_H_66_O_7_Na; *m*/*z* 645.4 [M + Na]^+^, ESI-MS) was determined to possess an adjacent bis-bis-tetrahydrofuran ring system located between C-15 and C-24 according to both MS and ^1^H-NMR data. The presence of the α, β-unsaturated γ-lactone was supported by peaks in the ^1^H-NMR spectrum at 7.18 (1H, d, *J* = 1.6, H-35), 5.06 (1H, dq, H-36), 3.85 (1H, m, H-4), 2.50 (1H, m, H-3a), 2.40 (1H, m, H-3b), and 1.44 (3H, d, *J* = 6.7, H-37), indicating the absence of a hydroxyl substitution at C-3/4/5 and confirmed by ^13^C-NMR data 174.33 (C-1), 151.77 (C-35), 131.12 (C-2), 77.96 (C-36), 69.99 (C-4), 33.2 (C-3), and 19.05 (C-37). Compound **7** (C_37_H_66_O_7_Na; *m*/*z* 645.4 [M + Na]^+^, ESI-MS) was determined to possess an adjacent bis-tetrahydrofuran ring system located between C-15 and C-24, according to both MS and NMR data. The occurrence of the α,β-unsaturated γ-lactone was indicated by the peaks in the ^1^H-NMR at 6.98 (1H, q, H-35), 4.99 (1H, qq, H-36), 2.25 (2H, t, H-3), and 1.40 (3H, d, H-37), indicating the presence of a hydroxyl substitution at C-34. Additionally, the spectrum revealed the presence of a terminal methyl group as a triplet at 0.87 (C-34). ^13^C-NMR data confirmed the existence of α,β-unsaturated γ-lactone by the presence of seven oxygenated carbons at 83.30 (C-16), 82.21 (C-19), 82.52 (C-20), and 82.78 (C-23). The remaining three hydroxylated carbons, C-15, C-24, and C-29, gave rise to peaks at 74.12, 71.39, and 71.7,7 respectively (Table 1).

Additionally, annonacin (**8**) and annonisin (**9**) were identified in the ethyl acetate extract of the leaves by the existence of product ions in the MSMS of [M + Li]^+^ *m*/*z* = 603 and 617, respectively. Both compounds **8** and **9** revealed intense peaks for fragments at *m*/*z* 491, 391, 305, 233, and 205, and 505, 433, 347, 275, and 205 respectively, and were previously been reported in *A. atemoya* seeds [18].

The most concentrated ACs in the leaves according to TLC analysis gave rise to a product ion [M + Li]^+^ (*m*/*z* = 645), leading to the molecular formula C_37_H_66_O_8_. indicating bullatanocin (**10**) and atemoyacin-E (**11**). A series of fragment ions at *m*/*z* 533, 363, 335, 277, and 205, and 533, 363, 293, 265, 207, and 149 in the MSMS confirmed the presence of compounds **10** and **11**, respectively. Both compounds have previously been reported in *A. atemoya* seeds [15,22]. For compound **10**, the presence of α,β-unsaturated γ-lactone was confirmed by peaks in the ^1^H-NMR at 7.18 (1H, q, H-35), 5.06 (1H, qq, H-36), 3.86 (1H, m, H-4), 2.54 (1H, m, H-3a), 2.40 (1H, m, H-3b), and 1.42 (3H, d, H-37). Peaks in the ^13^C-NMR at 174.46 (C-1), 151.90 (C-35), 131.11 (C-2), 78.01 (C-36), 69.88 (C-4), and 19.07 (C-37) confirmed the presence of α,β-unsaturated γ-lactone, and the presence of two nonadjacent THF rings was indicated by proton resonances at 3.86 (H-12) and 3.81 (H-15, 20, 23).

The other chemical constituents identified from the leaves of *A. atemoya* were alkaloids, with TLC and MS confirming their presence in the ethanolic extract. The positive ESI-MS *m*/*z*: 291 [M + H]+ indicated lysicamine (**12**), and the MS data were in agreement with those in the literature [1,39]. The other oxoaporphine alkaloid found in both ethyl acetate and ethanol extracts was lanuginosine (**13**), with a positive ESI-MS reported at *m*/*z*: 304 [M + H]^+^. The MS data agreed with those in the literature. Both compounds (**12** and **13**) were previously been reported in the leaves of *A. atemoya* [1]. Roemerine (**14**), an aporphine alkaloid, was identified in the ethanol extract with an ESI-MS *m*/*z*: 279 [M + H]^+^. This compound has not previously been reported in *A. atemoya* but has been reported in the leaves of *A. squamosa* and the stem of *A. cherimola* [7,40,41]. Finally, another aporphine alkaloid, corydine (**15**), was confirmed by ESI-MS *m*/*z*: 341 [M + H]^+^ and was previously reported in the leaves of *A. squamosa* [40], but not in *A. atemoya*.

#### 2.3.2. Pulp

The concentration of ACs in the pulp was very low compared with that in the leaves and seeds. However, the presence of ACs could be detected in all three extracts (hexane, ethyl acetate, and ethanol) by NMR and MS analysis. Compound **1** was identified in all three extracts of pulp, and the MSMS confirmed the presence of the product ion of [M + Li]^+^ (*m*/*z* = 537) and intense fragments at *m*/*z* 313, 301, 285, and 271. A compound identified only in the ethyl acetate extract of the pulp was montanacin A (**16**), which showed a peak in the ^1^H NMR at 7.20 ppm, and a [M + Li]^+^ (*m*/*z* = 647), leading to the molecular formula C_37_H_68_O_8_. The MSMS analysis revealed fragments at *m*/*z* 535, 463, 337, 277, and 205, suggesting the localisation of the mono-THF ring at C-20 and C-23. This compound was previously reported in *A. montana* seeds, and our result is consistent with those previously reported [42].

#### 2.3.3. Seeds

All three solvent extracts of the seeds contained similar ACs, with a greater diversity of compounds in the ethyl acetate extract. Compound **1** was found only in the hexane extract of the seeds with a molecular mass of 530 by ESI-MS ([M+H]^+^ *m*/*z* = 531). An adjacent bis-THF compound, desacetyluvaricin (**17**), was identified solely in the hexane extract, with a molecular mass determined by ESI-MS ([M+H]^+^ *m*/*z* = 606), leading to the molecular formula C_37_H_62_O_6_. The MSMS analysis revealed intense fragments at *m*/*z* 347, 275, 205, and 177, suggesting the localisation of a bis-THF ring between C-16 and C-23. This compound was previously reported in the seeds of *A. atemoya* [20]. Interestingly, nine compounds were common to the three extracts according to the MS and MSMS analyses. These compounds were identified as diepomuricanin A (**2**) and B (**3**), annotemoyin-1 (**4**) and -2 (**5**), squamocin G (**6**), squamocin C (**7**), bullatanocin (**10**), atemoyacin-E (**11**), and atemoyacin-A (**18**). The identification of compounds **2**, **3**, **4**, **5**, **6**, **7**, **10,** and **11**, was reported above.

**Figure 1:**
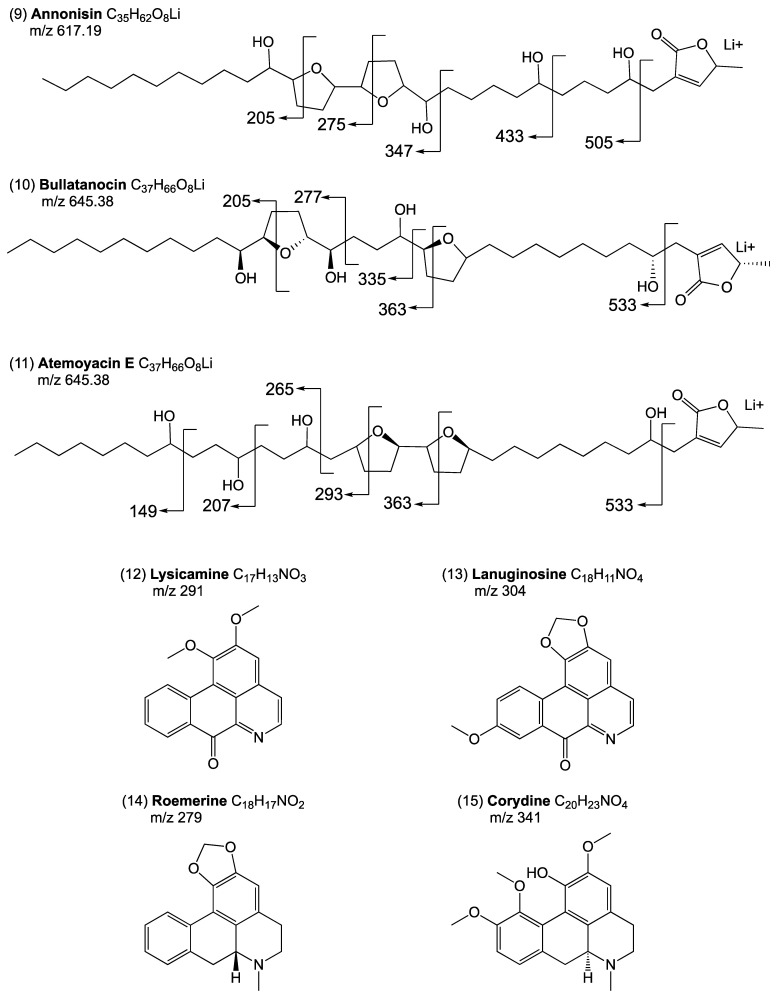

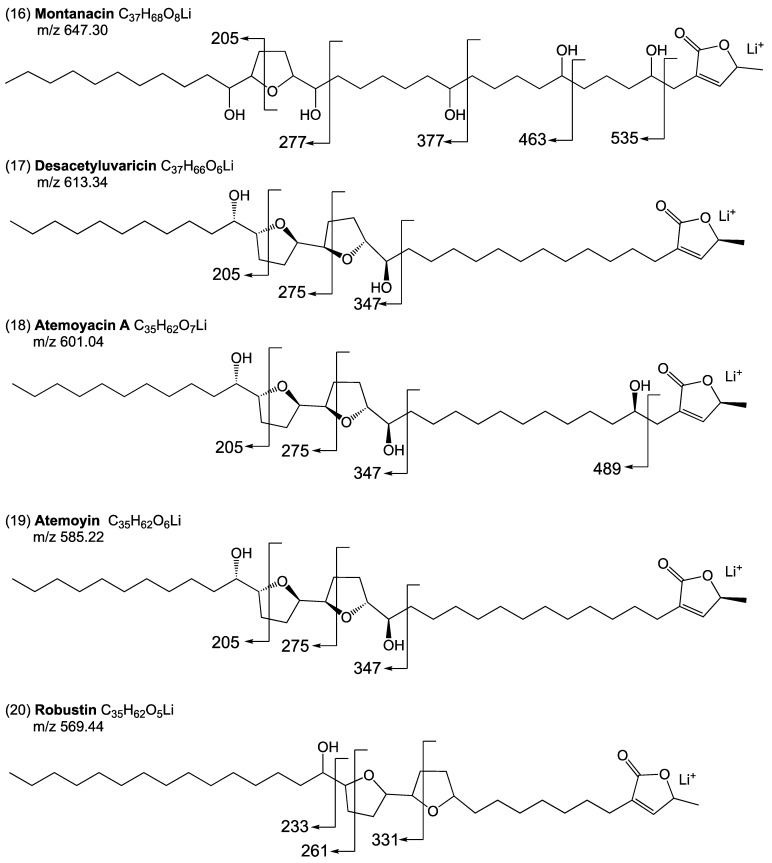
Structures and fragmentation patterns for the [M + Li]^+^ adducts of acetogenins and alkaloids identified in the leaves, pulp, and seeds of *A. atemoya*.

The molecular weight of compound **18** was determined as *m*/*z* 595 [MH^+^], corresponding to the molecular formula C_35_H_62_O_7_, and MSMS revealed a series of fragments at *m*/*z* 489, 347, 275, 205, and 177, confirming the presence of atemoyacin-A (**18**) in all three extracts. Atemoyacin-A (**18**) was previously reported in the seeds of *A. atemoya* Hort [16]. Additional compounds that were only identified in the ethyl acetate extract of the seeds were atemoyin (**19**) and robustocin (**20**). Compound **19** was determined to have a product ion of *m*/*z* = 601 [M + Na]^+^, and MSMS revealed intense fragments at *m*/*z* 347, 275, 205, and 177. The fragmentation pattern observed in the MSMS spectrum indicated that the two THF rings of (**19**) were located between C-13 and C-22. This compound was previously reported in the methanolic and ethanolic extracts of *A. atemoya* seeds [20]. Finally, compound (**20**) is reported for the first time in *A. atemoya,* with the MS showing a compound ion at *m*/*z* = 585 [M + Na]^+^. The MSMS revealed a fragmentation pattern with two intense fragments at *m*/*z* 261 and 233, confirming the occurrence of two THF rings between C-9 and C-18. This compound was previously reported only in the seeds of *A. muricata* [43]. The fragmentation patterns of each compound reported are shown in Figure 1. The NMR and MS spectra are shown in the Supplementary Materials (Figures S5–S26).

#### 2.3.4. KJ Cultivar

The second cultivar (KJ) was only studied by MS analysis, with some similarities noted between the cultivars. Compounds **1** and **8** were identified in the crude leaf extracts of both the AP and KJ cultivars. Compound **16**, however, was identified in the leaf extract of the KJ cultivar but was not detected in the leaves of the AP cultivar. Compound **1** was identified only in the ethanolic extract of the KJ cultivar fruit pulp, with a molecular mass of 530 determined by ESI-MS ([M+H]^+^ *m*/*z* = 531), leading to the molecular formula C_35_H_62_O_3_. Interestingly, although the extraction methods were identical for both cultivars, compound **1** was identified in all three solvent extracts of the AP cultivar pulp. The ESI-MS of the ethyl acetate extract of the KJ cultivar pulp indicated the presence of compounds **2** and **3**, with a molecular mass 564 *m/z*, corresponding to the molecular formula C_35_H_62_O_4_. Compound **8** was also found in both the hexane and ethanolic extracts of KJ fruit pulp, with a molecular weight determined by ESI-MS ([M+H]^+^ *m*/*z* = 596). However, compounds **2**, **3**, and **8** were not detected in the AP fruit pulp. Finally, compound **16** was found in both the hexane and ethyl acetate extracts of KJ pulp, with a molecular mass determined by ESI-MS ([M+H]^+^ *m*/*z* = 640). However, **16** was only detected in the ethyl acetate extract of the AP pulp.

The seeds of the KJ cultivar displayed similar ACs to those of the AP cultivar; however, ACs (**1**, **4**, **5**, and **17**–**20**) were not detected in any KJ seed extract. Two compounds, **8** and **16**, were found in all solvent extracts of the seeds from the KJ cultivar, with a molecular mass determined by ESI-MS ([M+H]^+^ *m*/*z* = 640 and 596, respectively) but were not detected in any extract of AP seeds. Additionally, compounds **2** and **3** found reported in both the hexane and ethyl acetate KJ seed extracts, with a molecular weight determined by ESI-MS ([M+H]^+^ *m*/*z* = 564), corresponding to the molecular formula C_35_H_62_O_4_. This is with AP, where they were detected in all seed extracts. Other compounds identified in the seeds of both cultivars were **6**, **7**, **10**, and **11**. However, these four ACs were identified only in the KJ ethyl acetate extract of the seeds, compared with the AP cultivar, which contained these compounds in all three solvent extracts of the seeds.

## 3. Materials and Methods

### 3.1. Plant Material Collection and Preparation

The fully matured fresh leaves, fruit, and seeds of *A. atemoya* cultivars (African Pride (AP) and KJ Pinks Mammoth (KJ)) were collected in July 2020, from a local farm in southeast Queensland, Australia. The leaves and fruits were washed with running tap water to remove dirt and debris. The leaves were spread out on a bench for air drying at room temperature in a well-ventilated area for one week. The seeds were removed from the fruit, washed, and air-dried. The fruit pulp was sliced and kept on the bench for air drying at room temperature for one month. The dehydrated leaves, pulp, and seeds were ground to a fine powder using a multifunctional grinder. The final weight of the leaf, pulp, and seed powders was 222.4 g, 380 g, and 50.6 g for the AP cultivar, and 180.9 g, 210 g, and 34.1 g for the KJ cultivar, respectively.

### 3.2. Preparation of Plant Extracts

The dried and powdered leaves, pulp, and seeds of both cultivars were initially macerated with hexane (2.5 L) and filtered. The residues were then extracted with ethyl acetate (2.5 L) and filtered. Finally, the residue was extracted with ethanol (2.5 L). All extractions were carried out once at room temperature with stirring for 72 h and filtrated using vacuum filtration. After each extraction, the solvents were evaporated under reduced pressure, affording the yields shown in Table 2.

### 3.3. Chemical and Reagents

Laboratory-grade hexane, dichloromethane (DCM), ethyl acetate (EtOAc), ethanol (EtOH), and methanol (MeOH) were obtained from the School of Chemistry, University of Sydney. HPLC-grade methanol, acetonitrile, LCMS-grade methanol, and deuterated solvents (CD_3_OD (99.8% D) and CDCl_3_ (99.8% D) were purchased from Sigma-Aldrich (Sydney, Australia).

### 3.4. Phytochemical Screening

Each of the crude hexane, ethyl acetate, and ethanol extracts of the three plant parts was analysed by TLC in order to investigate their phytochemical constituents. A total of 2 mg of each extract was dissolved in 1 mL of solvent and spotted on a TLC plate, which was subsequently developed in a CAMAG 20 × 10 cm twin-through glass developing chambers using MeOH:DCM (10:90 *v*/*v*) as the mobile phase. The TLC plate was then examined under UV light at 254 nm and 365 nm; then, the TLC plate was further visualised using Kedde’s reagent to identify ACs. Firstly, the TLC plate was dipped in a 2% solution of 3,5-dinitrobenzoic acid in 100 mL of ethanol and left to dry for five minutes and then sprayed with 5% KOH in 100 mL of ethanol. For alkaloid identification, TLC plates were visualised with Dragendorrf’s reagent.

### 3.5. TLC and CC

Analytical plates for TLC were silica gel 60 F_254_, aluminium sheets, 200 -µm, (20 × 20 cm), and Merck silica gel 60 (particle size 0.040–0.063 mm) (Merck KGaA, Darmstadt, Germany). A few milligrams of each crude extract of the leaves, pulp, and seeds dissolved in methanol was spotted on the TLC plate and eluted using methanol:dichloromethane (10:90) as a mobile phase. Plates were visualised under ultraviolet light (UV) at 254 nm and 365 nm, and in order to visualise the compounds, Kedde’s (acetogenins) and Dragendorff’s (alkaloids) reagents were used. Kedde’s reagent was prepared as solutions of 2% (2 g) of 3.5-dinitrobenzoic acid in 100 mL of ethanol and 5% (5 g) of KOH in 100 mL of ethanol [44]. Dragendorff’s reagent was prepared as follows: solution A: 1.7 g of bismuth nitrate was dissolved in 100 mL of water:acetic acid (4:1). Solution B: 40 g of potassium iodide was dissolved in 100 mL of water. Solutions A and B were combined as follows: 5 mL A + 5 mL B + 20 mL acetic acid + 70 mL water.

### 3.6. Flash Chromatography

All purifications by flash chromatography were performed on a Reveleris iES system (W. R. Grace & Co.-Conn., Columbia, MD, USA) using the Reveleris**^®^** Navigator^™^ software. This instrument consists of a binary pump with four solvent selection, fraction collector and evaporating light scattering detector (ELSD). Reveleris**^®^** flash cartridges packed silica.

### 3.7. NMR

All the fractions obtained and the isolated compounds were characterised by ^1^H-NMR and ^13^C-NMR spectroscopy. ^1^H and ^13^C spectra were recorded at 400 MHz and 100 MHz, respectively, using a Varian Gemini 400-MR automated spectrometer (Agilent Technologies, Santa Clara, CA, USA) at room temperature, using CDCl_3_ as solvent referenced to the residual signal set at 7.26 ppm for ^1^H and 77.0 ppm for ^13^C.

### 3.8. MS and MS/MS

Mass spectra were carried out on a Thermo Scientific TSQ series Quantum Access Max liquid chromatography mass spectrometry (LC-MS/MS) system (Thermo Fisher Scientific, Waltham, MA, USA), using heated electrospray ionisation in positive ionisation mode. High-purity nitrogen (BOC, Sydney, Australia) at 60 psi was used as the sheer gas, and ultra-high purity argon (BOC) at 2 mTorr was used as the collision gas. The mass spectroscopy was conducted at a voltage of 4500 V. The heated capillary and vaporiser were heated at 380 °C and 350 °C, respectively. The mass spectra were acquired at a scan range of 100–1000 m/z. Data were analysed with XCalibur 4.1.50 (Thermo Fisher Scientific, Waltham, MA, USA) software. For the MS/MS experiment, a solution of crude extracts was prepared at a concentration of 1 mg/mL in MeOH, and the injection volume was 5 µL. The optimal collision energy was 70 eV, and lithium iodide (2 mM in MeOH) was added to each sample before analysis.

### 3.9. Isolated Compounds

#### 3.9.1. Squamocin G (**6**)

White powder ^1^H-NMR (400 MHz, CDCl_3_) 0.87 (3H, t, *J* = 6.9 Hz, H-34), 1.44 (3H, d, *J* = 6.7 Hz, H-37), 2.40 (1H, dd, *J* = 15.0, 8.2 Hz, H-3a), 2.50 (1H, br d, *J* = 15.0 Hz, H-3b), 3.39 (1H, m, H-15), 3.84–3.93 (3H, m, H-16, -23, -24), 3.85 (1H, m, H-4), 3.84–3.90 (2H, m, H-19, 20), 5.06 (1H, qq, *J* = 6.8, 1.4 Hz, H-36), 7.18 (1H, br s, H-35), ^13^C-NMR (400 MHz, CDCl_3_) 14.10 (C-34), 19.10 (C-37), 22.67 (C-33), 24.49 (C-22), 25.54 (C-6 or C-13), 25.61 (C-13 or C-6), 26.03 (C-26), 28.34 (C-17), 28.88–28.93 (C-18, C-21), 29.10–29.67 (C-7, C-8, C-9, C-10, C-11, C-12, C-27, C-28, C-29, C-30, C-31), 31.89 (C-32), 32.42 (C-25), 33.2 (C-3), 33.32 (C-14), 37.39 (C-5), 69.99 (C-4), 71.33 (C-24), 74.04 (C-15), 77.97 (C-36), 82.26 (C-19 or C-20), 82.51 (C-20 or C-19), 82.77 (C-23), 83.2 (C-16), 131.18 (C-2), 151.77 (C-35), 174.33 (C-1).

#### 3.9.2. Squamocin C (**7**)

White powder ^1^H-NMR (400 MHz, CDCl_3_) 0.87 (3H, t, *J* = 6.7 Hz, H-34), 1.40 (3H, d, *J* = 7.1 Hz, H-37), 2.25 (2H, t, *J* = 7.7 Hz, H-3), 3.40 (1H, m, H-15), 3.59 (1H, m, H-29), 3.82–3.96 (5H, m, H-16, -19, -20, -23, -24), 4.99 (1H, q, *J* = 6.9 Hz, H-36), 6.98 (1H, s, H-35), ^13^C-NMR (400 MHz, CDCl_3_) 14.1 (C-34), 19.2 (C-37), 22.0 (C-26), 22.6 (C-33), 24.80 (C-22), 25.15 (C-3), 25.31 (C-31), 25.64 (C-13, C-27), 27.37 (C-4), 28.36 (C-17), 28.9 (C-18, -21), 29.16 (C-5), 29-30 (C-6, C-7, C-8, C-9, C-10, C-11, C-12), 31.9 (C-32), 32.45 (C-25), 33.28 (C-14), 37.23 (C-30), 37.48 (C-28), 71.39 (C-24), 71.77 (C-29), 74.12 (C-15), 77.4 (C-36), 82.21 (C-19), 82.52 (C-20), 82.78 (C-23), 83.3 (C-16), 134.3 (C-2), 148.87 (C-35), 173.9 (C-1).

## 4. Conclusions

Several *Annona* species have been used as traditional medicines, particularly across South America and Asia, and have been phytochemically well characterised. Many of the ACs present in *Annona* species have also been shown to have anticancer activities. *A. atemoya* is a hybrid of two *Annona* species: *A. squamosa* (sugar apple) and *A. cherimola* (Cherimoya), first reported in 1907, which is gaining importance as a commercially important crop due to its creamy and sweet fruit. However, only few studies have investigated the phytochemistry of *A. atemoya*. Some previous studies have reported the presence of ACs in the seeds of *A. atemoya* [16,17,18,20,21,22]; our data are the first to show that ACs are also present in the leaves and pulp of *A. atemoya*.

In total, twenty compounds were identified: sixteen ACs (**1**–**11** and **16**–**20**) and four alkaloids (**12**–**15**). The seeds of the AP cultivar were the richest in ACs, with fourteen compounds identified (**1**–**7**, **10**, **11**, **17**–**20**) in all three extracts, compared with the pulp, which only contained two ACs (**1** and **16**). In addition, the ethyl acetate crude extracts of both the leaves and seeds were richer in ACs in comparison with the other crude extracts. Compounds **6** and **7** were isolated, and their structures and absolute configuration were determined through the interpretation of the spectroscopic data and comparison with previously reported data. Montanacin A (**16**) is reported for the first time in the pulp of the AP cultivar and the leaves of the KJ cultivar of *A. atemoya*.

Our results demonstrated that *A. atemoya* contains a range of acetogenins and alkaloids, similar to those found in other *Annona* species. To the best of our knowledge, this is the first comprehensive study of the ACs in the leaves and pulp of *A. atemoya*. Although the two cultivars contained similar ACs, the KJ cultivar contained only 9 ACs compared with the 16 detected in the AP cultivar, indicating that further study of other cultivars should be undertaken. Given the increasing consumption of custard apples, their potential health benefits, and the toxicity of some of the constituents found in the fruit and leaves, a study to quantify some of the relevant constituents is also warranted.

## Figures and Tables

**Table 1:** ^1^H and ^13^C NMR data for compounds **6** and **7** recorded in CDCl_3_.

	6		7	
**Position**	**δ H (*J* Hz)**	**δ C**	**δ H (*J* Hz)**	**δ C**
**1**		174.33		173.9
**2**		131.18		134.3
**3**	2.40, 2.50	33.2	2.25	25.15
**4**	3.85	69.99	1.52	27.37
**5**	1.42	37.39	1.2–1.7	29.16
**6**	1.25–1.4	25.54	1.2–1.7	29–30
**7**	1.25–1.4	29.10–29.67	1.2–1.7	29–30
**8**	1.25–1.4	29.10–29.67	1.2–1.7	29–30
**9**	1.25–1.4	29.10–29.67	1.2–1.7	29–30
**10**	1.25–1.4	29.10–29.67	1.2–1.7	29–30
**11**	1.25–1.4	29.10–29.67	1.2–1.7	29–30
**12**	1.25–1.4	29.10–29.67	1.2–1.7	29–30
**13**	1.25–1.4	25.61	1.2–1.7	25.64
**14**	1.33–1.56	33.32	1.2–1.7	33.28
**15**	3.39	74.04	3.40	74.12
**16**	3.84–3.93	83.2	3.82–3.96	83.3
**17**	1.97–1.63	28.34	1.52–1.9	28.36
**18**	1.97–1.63	28.88	1.52–1.9	28.9
**19**	3.93	82.51	3.82–3.96	82.21
**20**	3.84	82.26	3.82–3.96	82.52
**21**	1.97–1.63	28.93	1.52–1.9	28.9
**22**	1.92–1.90	24.49	1.52–1.9	24.80
**23**	3.84–3.93	82.77	3.82–3.96	82.78
**24**	3.84–3.93	71.33	3.82–3.96	71.39
**25**	1.33–1.56	32.42	1.25–1.4	32.45
**26**	1.25–1.4	26.03	1.25–1.4	22.0
**27**	1.25–1.4	29.10–29.67	1.25–1.4	25.64
**28**	1.25–1.4	29.10–29.67	1.25–1.4	37.48
**29**	1.25–1.4	29.10–29.67	3.59	71.77
**30**	1.25–1.4	29.10–29.67	1.25–1.4	37.23
**31**	1.25–1.4	29.10–29.67	1.25–1.4	25.31
**32**	1.25–1.4	31.89	1.25–1.4	31.9
**33**	1.25–1.4	22.67	1.25–1.4	22.6
**34**	0.87	14.10	0.78	14.1
**35**	7.18	151.77	6.98	148.87
**36**	5.06	77.97	4.99	77.4
**37**	1.44	19.10	1.40	19.2

**Table 2:** The final weight of crude extracts of *A. atemoya* cultivars.

	AP Cultivar			KJ Cultivar		
	Leaves (g)	Pulp (g)	Seed(g)	Leaves (g)	Pulp (g)	Seed (g)
**Extraction Solvent**						
Hexane	12.3	2.63	10.93	10	1.787	6.32
Ethyl acetate	3.9	0.783	1.72	3.2	0.884	1.3
Ethanol	24.7	25.52	1.5	22.48	30.2	1.09

## Data Availability

Not applicable.

## Supplementary Materials

The following supporting information can be downloaded at: https://www.mdpi.com/article/10.3390/ijms24032294/s1.
