# Supplementary material for: Identification of Annonaceous Acetogenins and Alkaloids from the Leaves, Pulp, and Seeds of Annona atemoya
Source: Int J Mol Sci. 2023 Jan 24;24(3):2294. doi: 10.3390/ijms24032294 (PMC9916654; doi:10.3390/ijms24032294)
Supplement: Supplementary file 1 [file ijms-24-02294-s001.zip › ijms-2102348-supplementary.pdf]

Identification of Annonaceous Acetogenins and Alkaloids from the Leaves, Pulp and Seeds of Annona Atemoya

Bassam S. M. Al Kazman[1], Joanna E. Harnett[1], Jane R. Hanrahan[1,*]

[a]The School of Pharmacy, Faculty of Medicine and Health, The University of Sydney, Camperdown, NSW 2006, Australia.

Supporting Information

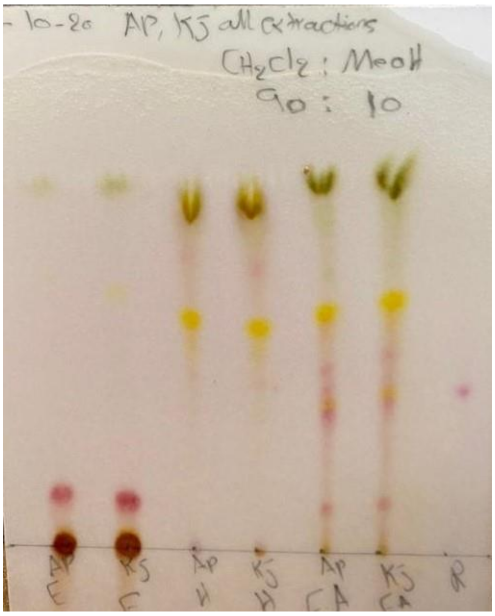

Figure S1: TLC profile of total *A. atemoya* (African Pride (AP), KJ Pinks Mammoth (KJ)) leaves, from left to right ethanolic extract, hexanolic extract and ethyl acetate extract and (R) (annonacin), indicated acetogenins as pink spot after spraying with Kedde's reagents.

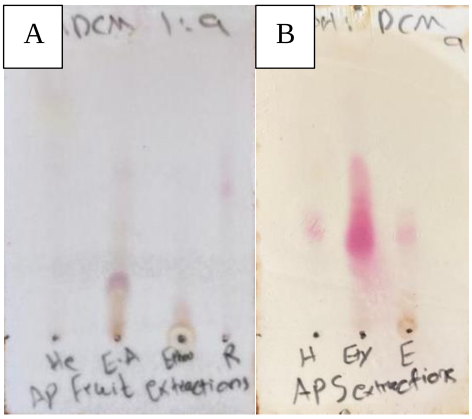

Figure S2: TLC profile of total *A. atemoya* (African Pride (AP)) pulp (A) and seeds (B), after spraying with Kedde's reagents.

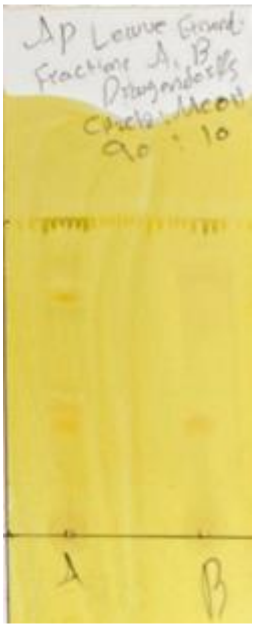

Figure S3: TLC plats for *A. atemoya* leaves ethanolic fraction (A) and (B) indicated alkaloids as orange spots after spraying with Dragendorff's reagent

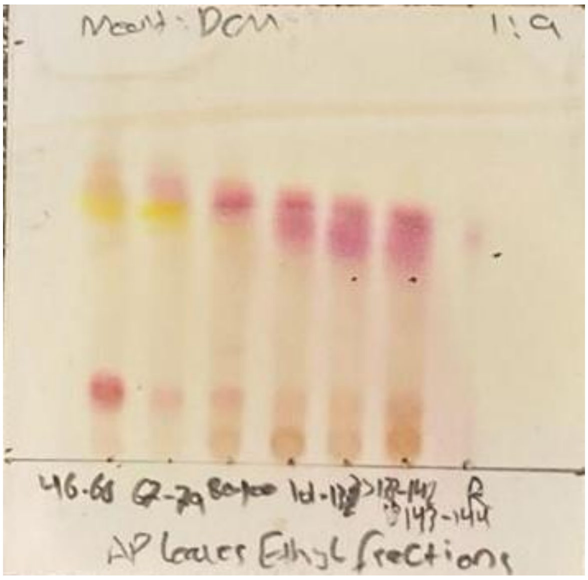

Figure S4: TLC plates for *A. atemoya* leaves ethyl acetate fractions (46 – 144) after spraying with Kedde's reagent

Table S1 *A. atemoya* leaves extract was submitted for flash chromatography experiments using different eluting systems

Extraction	Fractions indicated acetogenins with major contamination	Eluting system	Sub-fractions indicating acetogenins	Eluting system	Sub-fractions indicating acetogenins
**Hexane extract**	Fraction 1H (110 - 129) (145.2 mg)	(DCM: MeOH) (100: 0 v/v) to (DCM: MeOH) (0: 100 v/v)	Sub-fractions 1Ha (44 – 46) (39.8 mg)	(Hexane: EtOAc) (100: 0 v/v) to (Hexane: EtOAc) (0: 100 v/v)	Sub-fraction 1Hb 43 (1.5 mg), 44 (3.5 mg), 45 (4.3 mg) and 46 (0.8 mg)
	Fraction 2H (130 - 131) (33.3 mg)	(DCM: EtOH) (100: 0 v/v) to (DCM: EtOH) (0: 100 v/v)	Sub-fractions 2Ha (18 – 21) (3 mg)	Due to the small amount, these were submitted directly for MS analysis	
	Fraction 3H (138 - 143) (24 mg)	(DCM: EtOH) (100: 0 v/v) to (DCM: EtOH) (0: 100 v/v)	Sub-fractions 3Ha (35 – 38) (3.4 mg)		
**Ethanol extract**	Fraction B (80 mg)	(DCM: MeOH) (100: 0 v/v) to (DCM: MeOH) (0: 100 v/v)	Subfractions 37 to 43 (18 mg)	(DCM: EtOAc) (100: 0 v/v) to (DCM: EtOAc) (0: 100 v/v)	from 16 to 28 (1.4 mg) and from 30 to 36 (1.8 mg)
**Ethyl acetate extract**	Fraction 1E (46 – 66) (121.4 mg)	(DCM: MeOH) (100: 0 v/v) to (DCM: MeOH) (0: 100 v/v)	Sub-fractions 1Ea (37 - 44) (12.2 mg), and sub-fractions 1Eb (45 – 55) (6 mg)	(DCM: EtOAc) (100: 0 v/v) to (DCM: EtOAc) (0: 100 v/v)	Sub-fraction 1Ec (71 – 72) (9 mg)
	Fraction 2E (67 - 79) (66.7 mg)	(DCM: EtOAc) (100: 0 v/v) to (DCM: EtOAc) (0: 100 v/v)	Sub-fractions 2Ea (25) (44.3 mg)	(100% EtOAc)	Sub-fraction 2Eb (6 – 9) (11.2 mg)
	Fraction 3E (80 – 100) (119.4 mg)	(DCM: MeOH) (100: 0 v/v) to (DCM: MeOH) (0: 100 v/v)	Sub-fractions 3Ea (32 – 37) (73.9 mg)	(100% EtOAc)	Sub-fraction 3Eb (26 – 30) (9 mg) and Sub-fraction 3Ec (32 – 36) (4.7 mg)
	Fraction 4E (101 - 132) (164.2 mg)	(DCM: MeOH) (100: 0 v/v) to (DCM: MeOH) (0: 100 v/v)	Sub-fractions 4Ea (31 – 38) (65.4 mg)	(DCM: EtOAc) (100: 0 v/v) to (DCM: EtOAc) (0: 100 v/v)	Sub-fraction 4Eb (42 – 47) (30 mg)
	Fraction 5E (133 - 144) (190.9 mg)	(DCM: MeOH) (100: 0 v/v) to (DCM: MeOH) (0: 100 v/v)	Sub-fractions 5Ea (27 – 39) (73 mg)	(DCM: EtOAc) (100: 0 v/v) to (DCM: EtOAc) (0: 100 v/v)	Sub-fraction 5Eb (31 – 32) (8.6 mg)

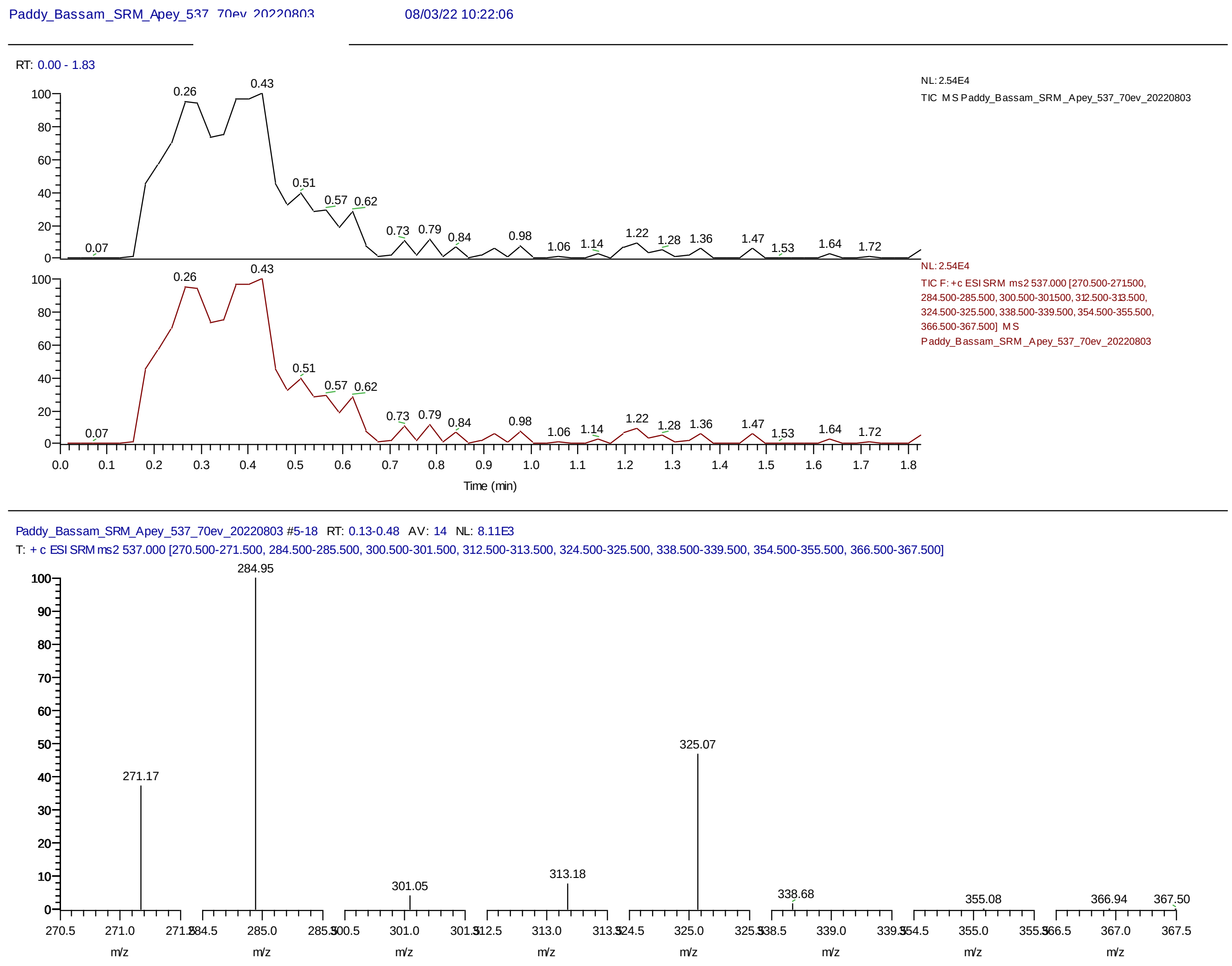

Figure S5: MSMS fragments for Epoxymurin-A

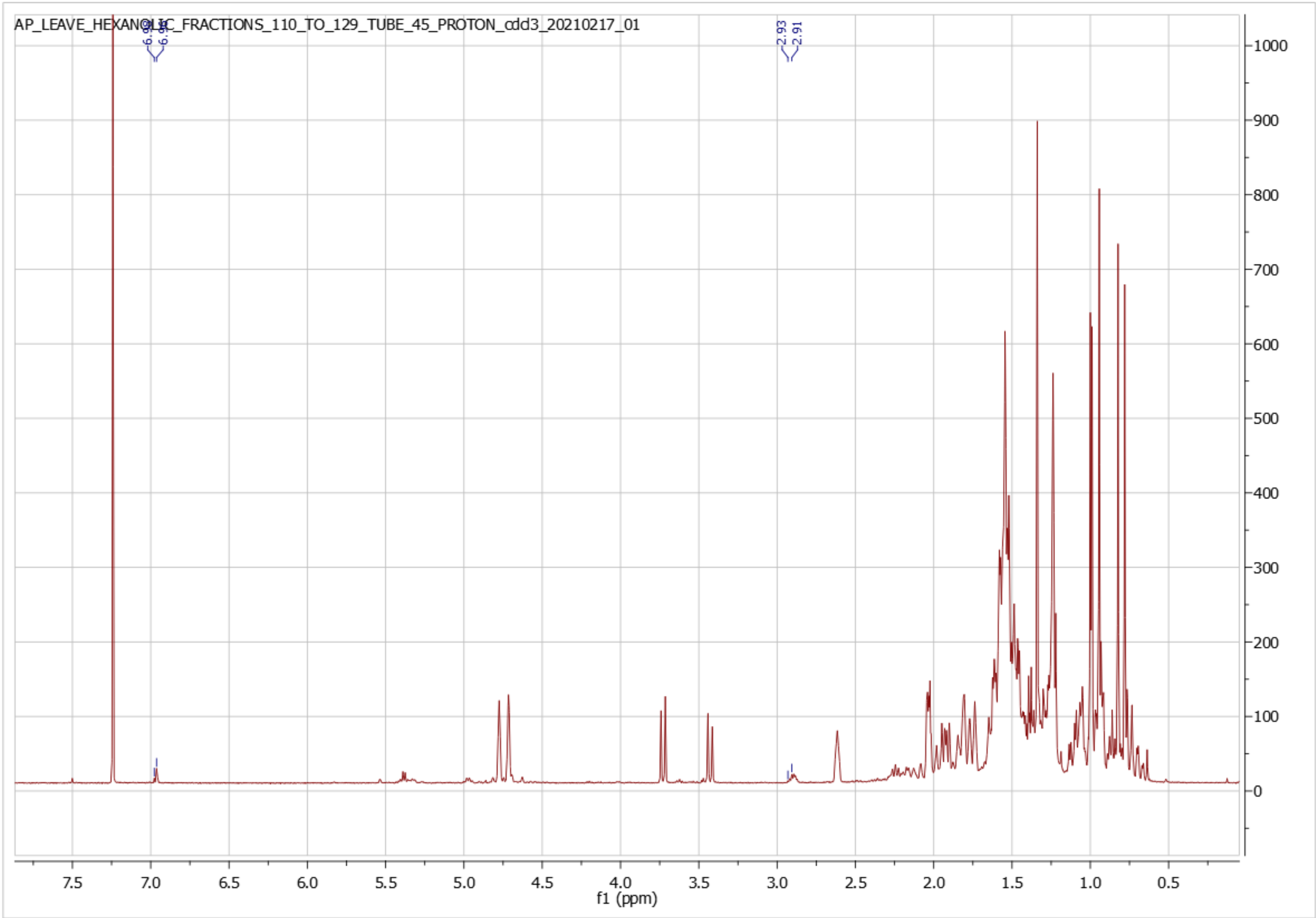

Figure S6: ^{1}H NMR [$CDCl_3$, 400 MHz], chemical shift for hexane extract

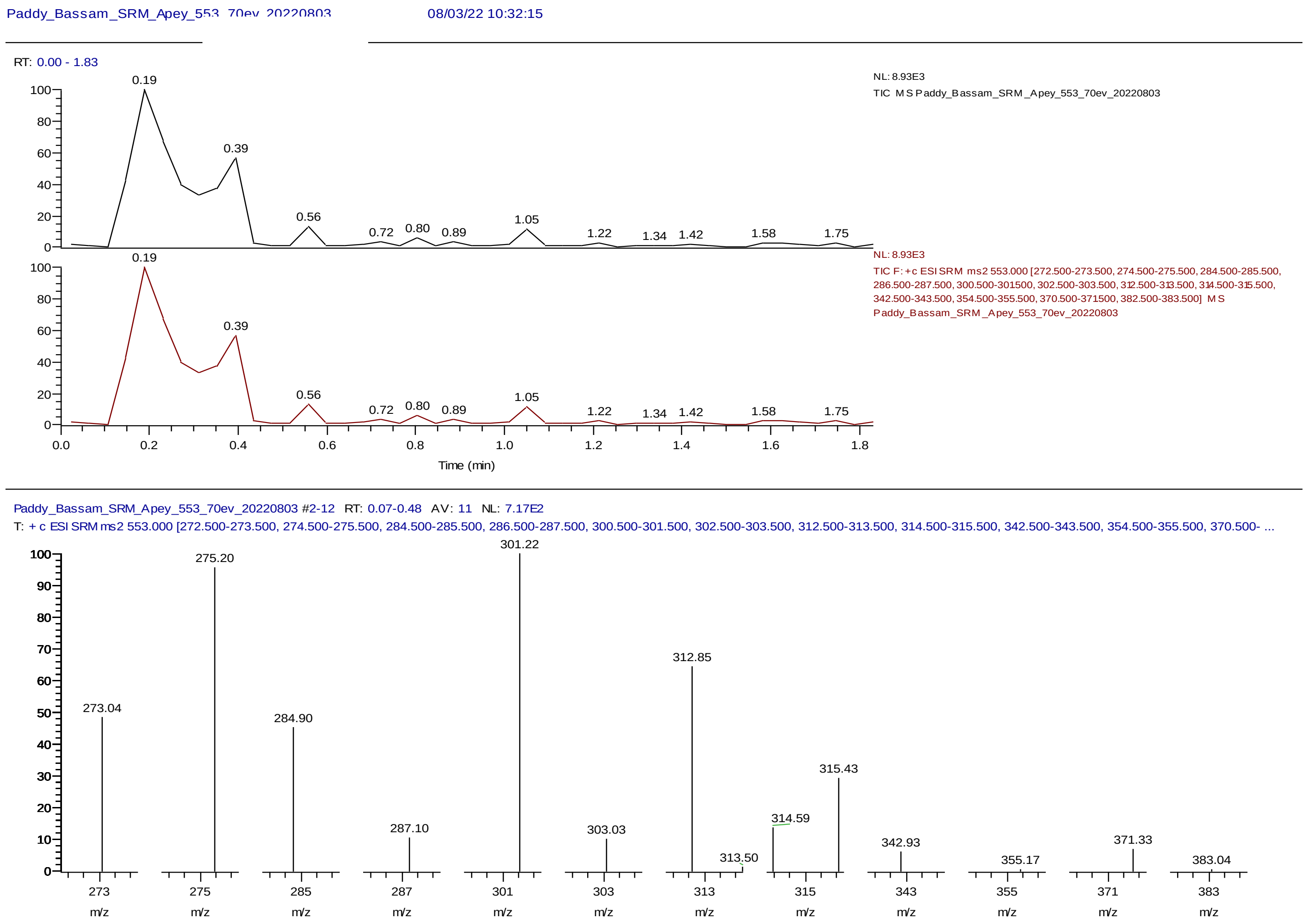

Figure S7: MSMS fragments for Diepomuricanin A and B

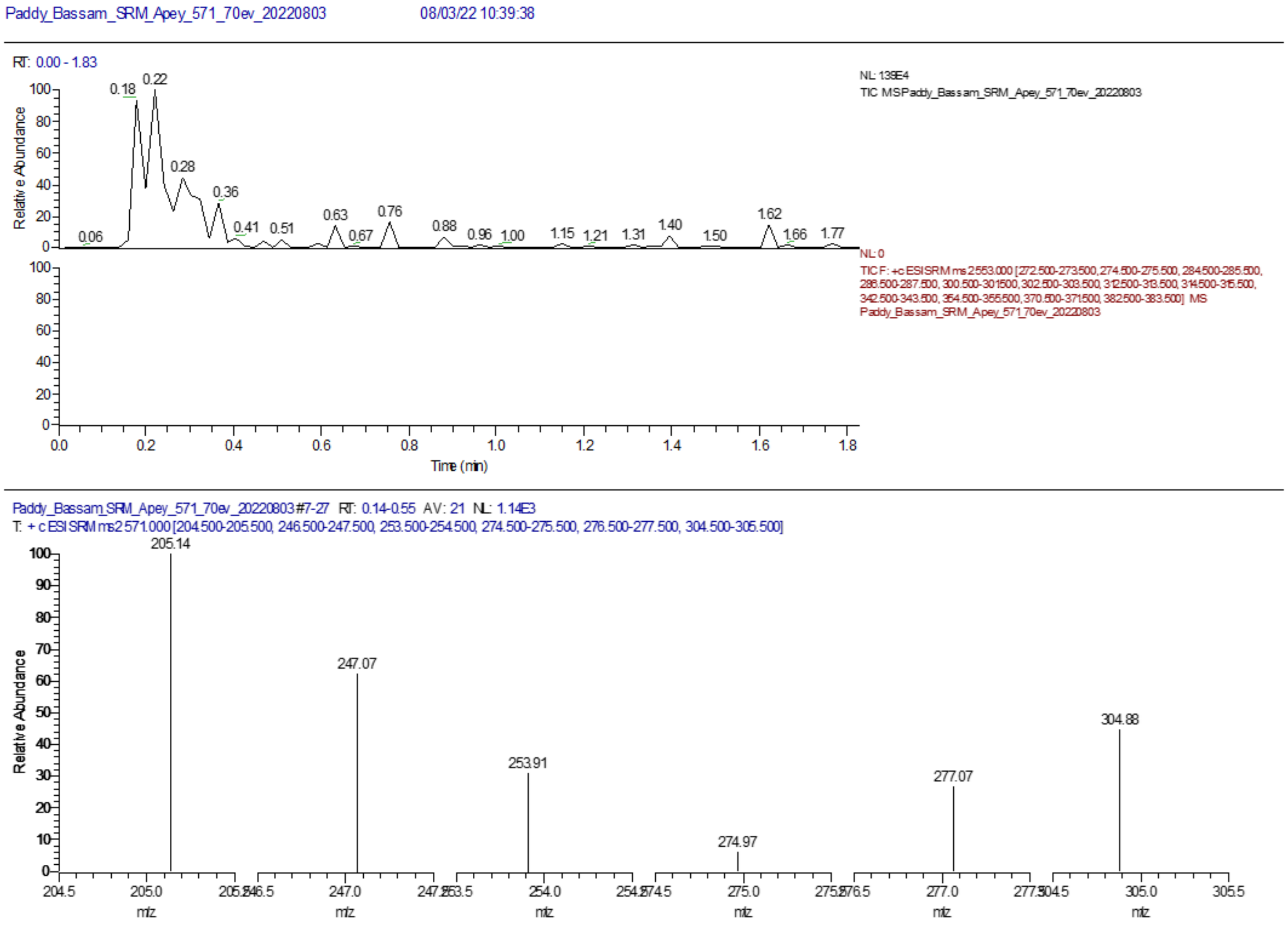

Figure S8: MSMS fragments for Annotemoyin-1 & 2

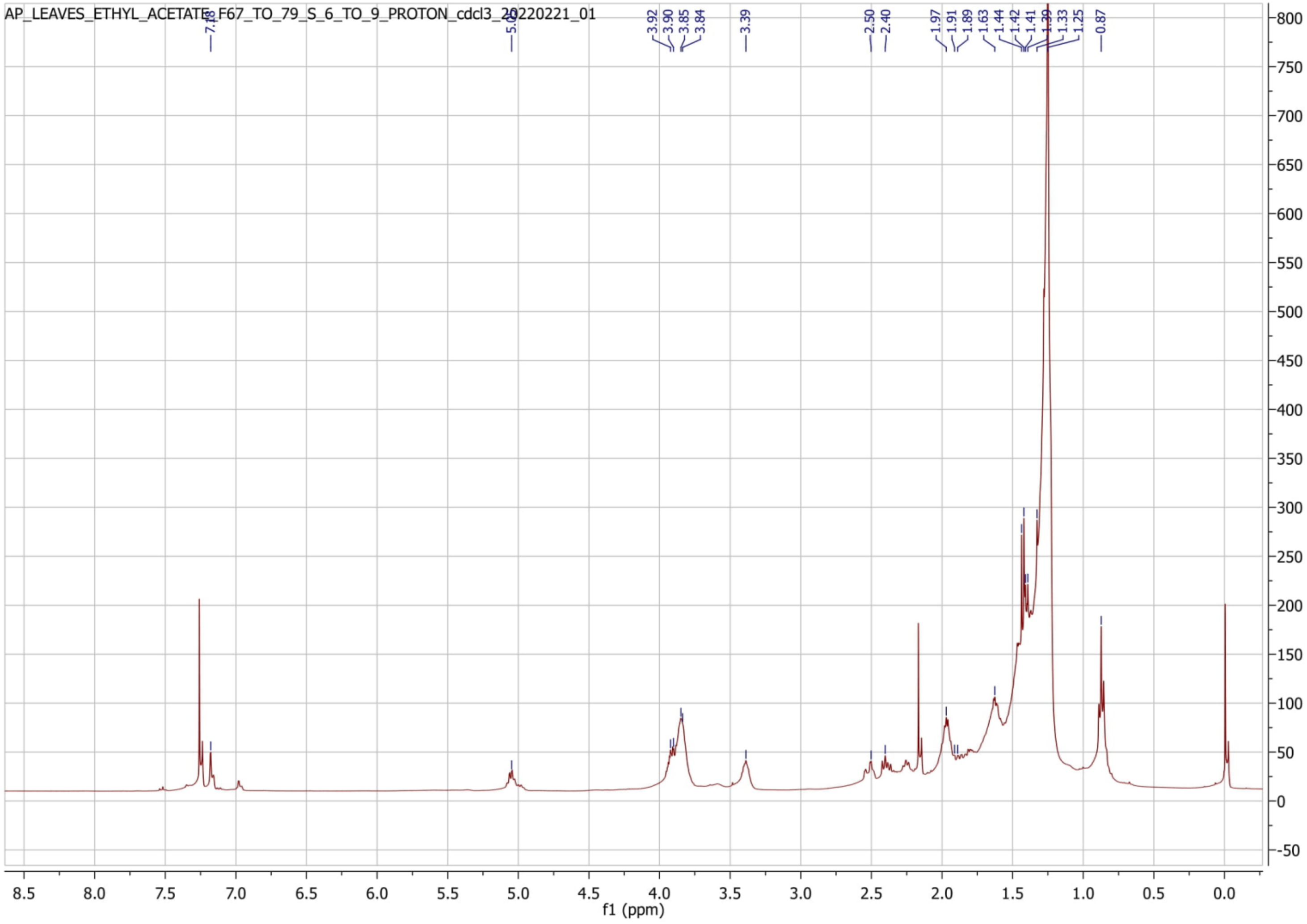

Figure S9: ^{1}H NMR [$CDCl_3$, 400 MHz], chemical shift for Squamocin G

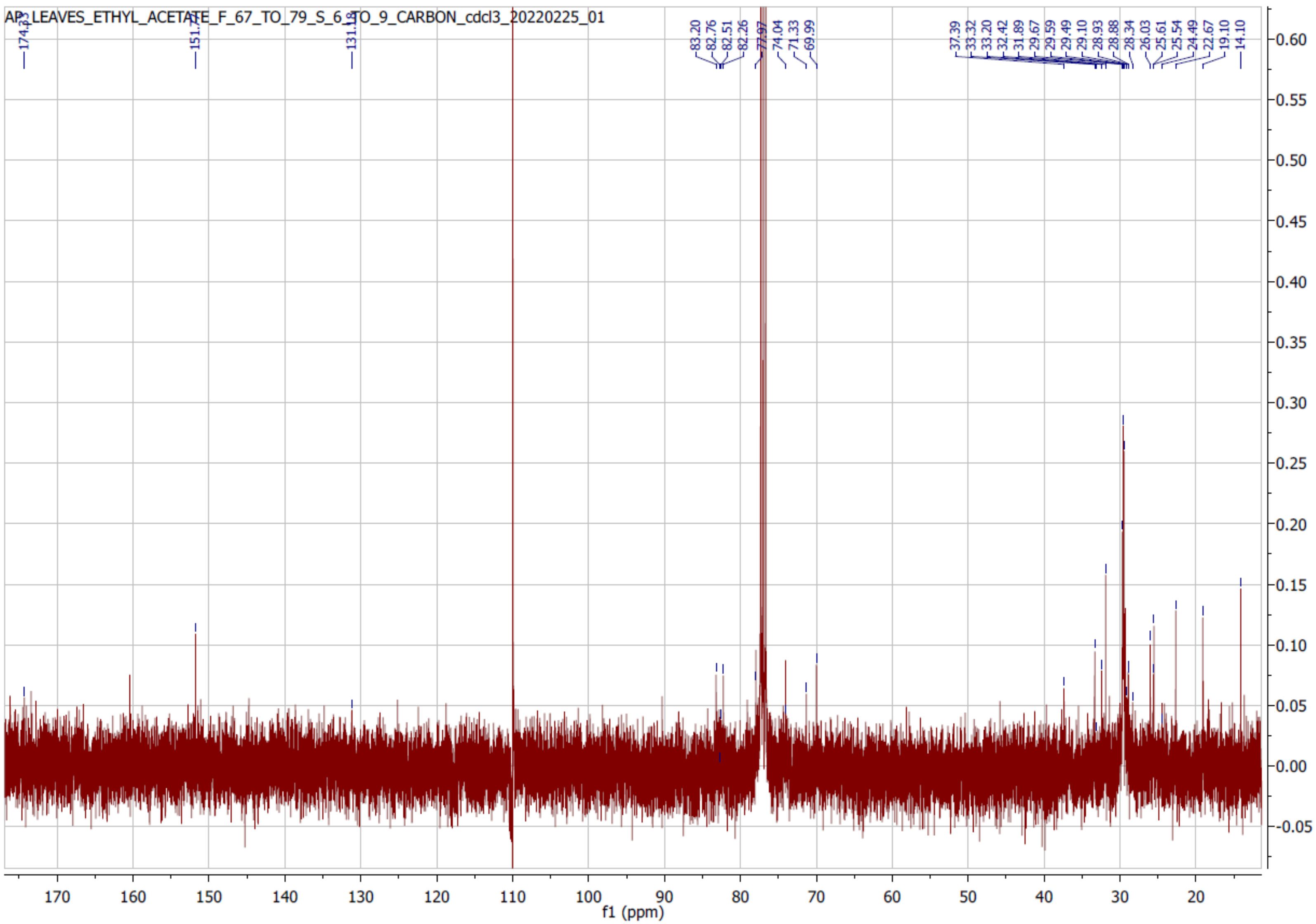

Figure S10: ^{13}C NMR [$CDCl_3$, 400 MHz], chemical shift for Squamocin G

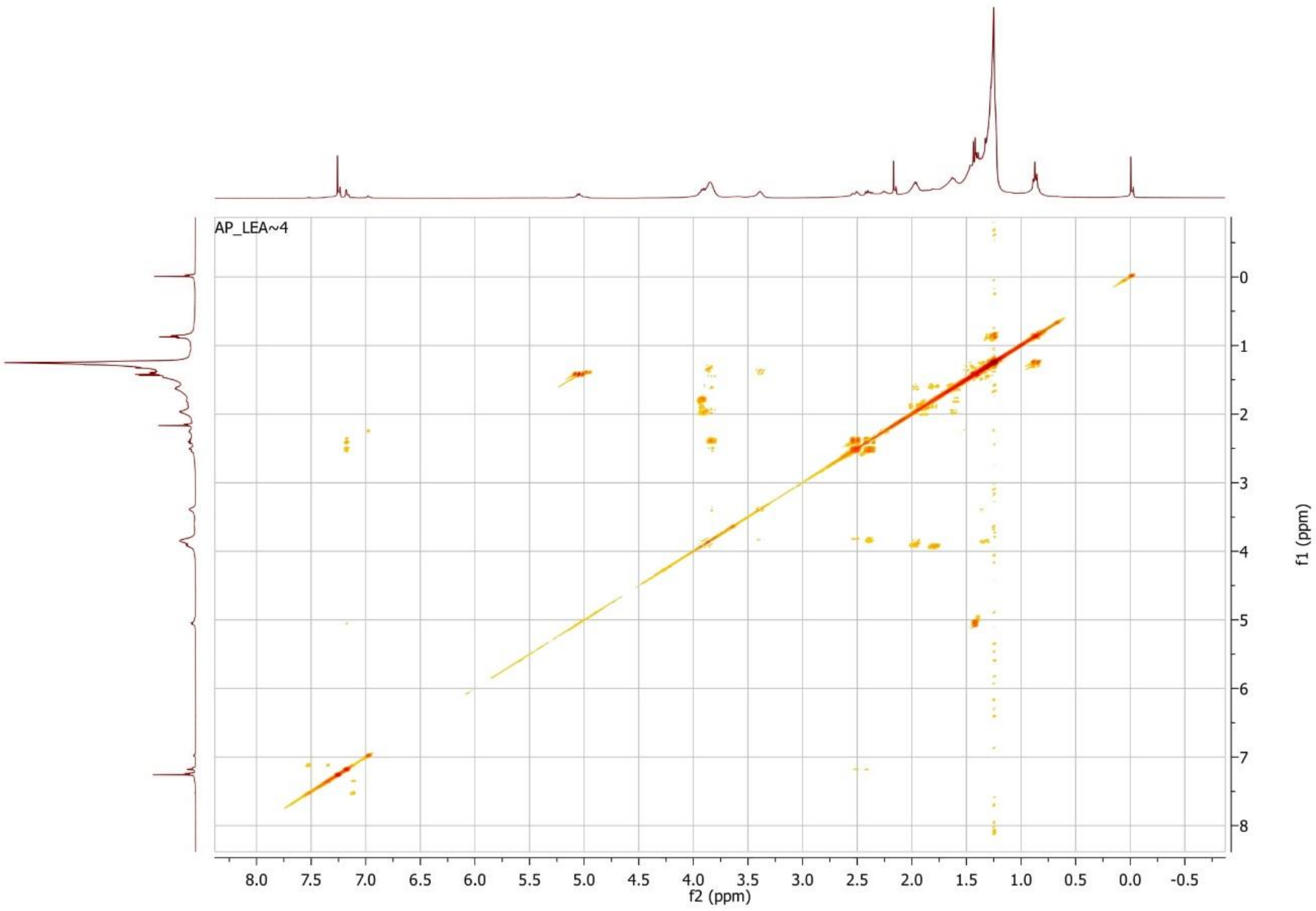

Figure S11: COSY correlation for Squamocin G

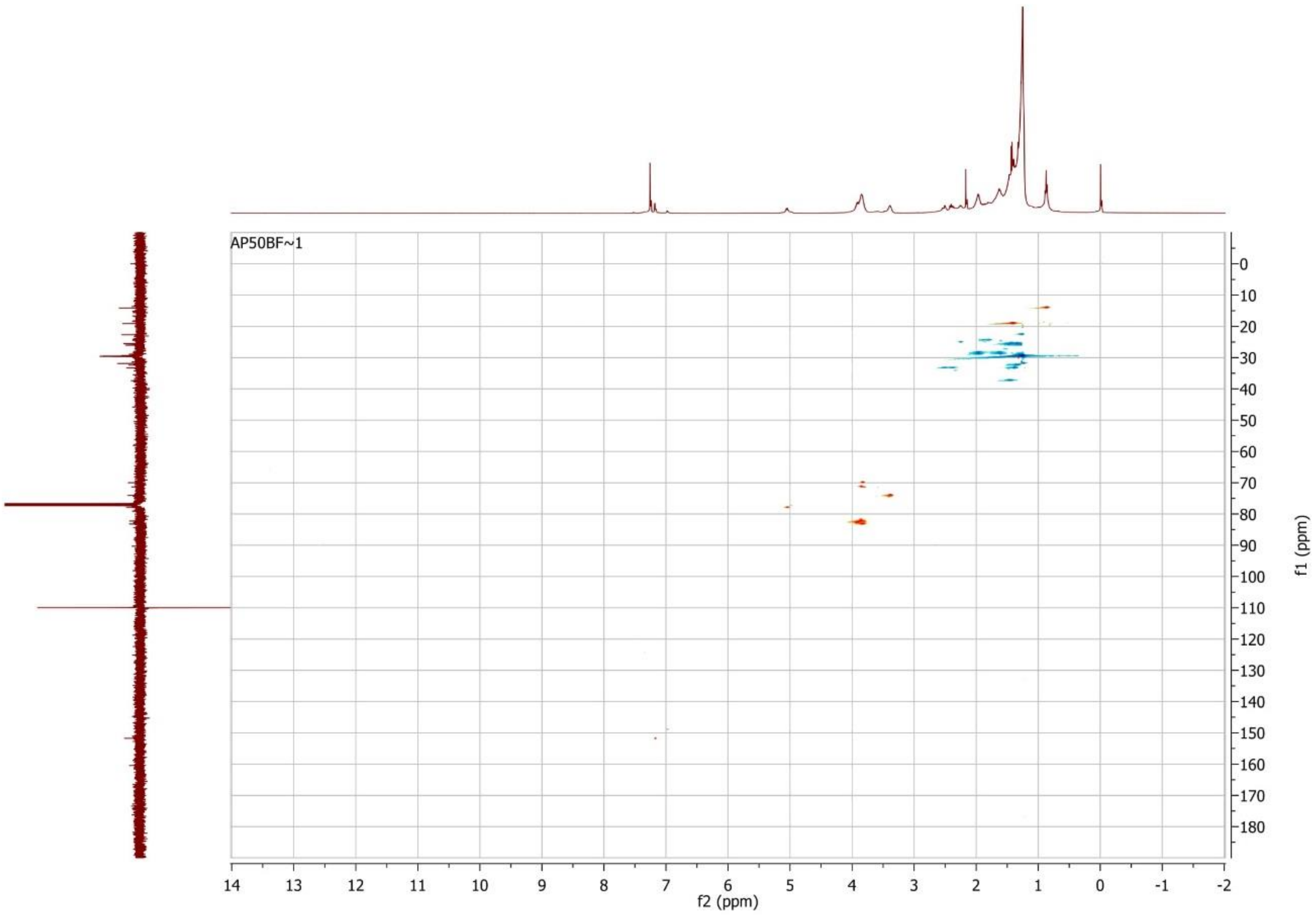

Figure S12: HSQCAD correlation for Squamocin G

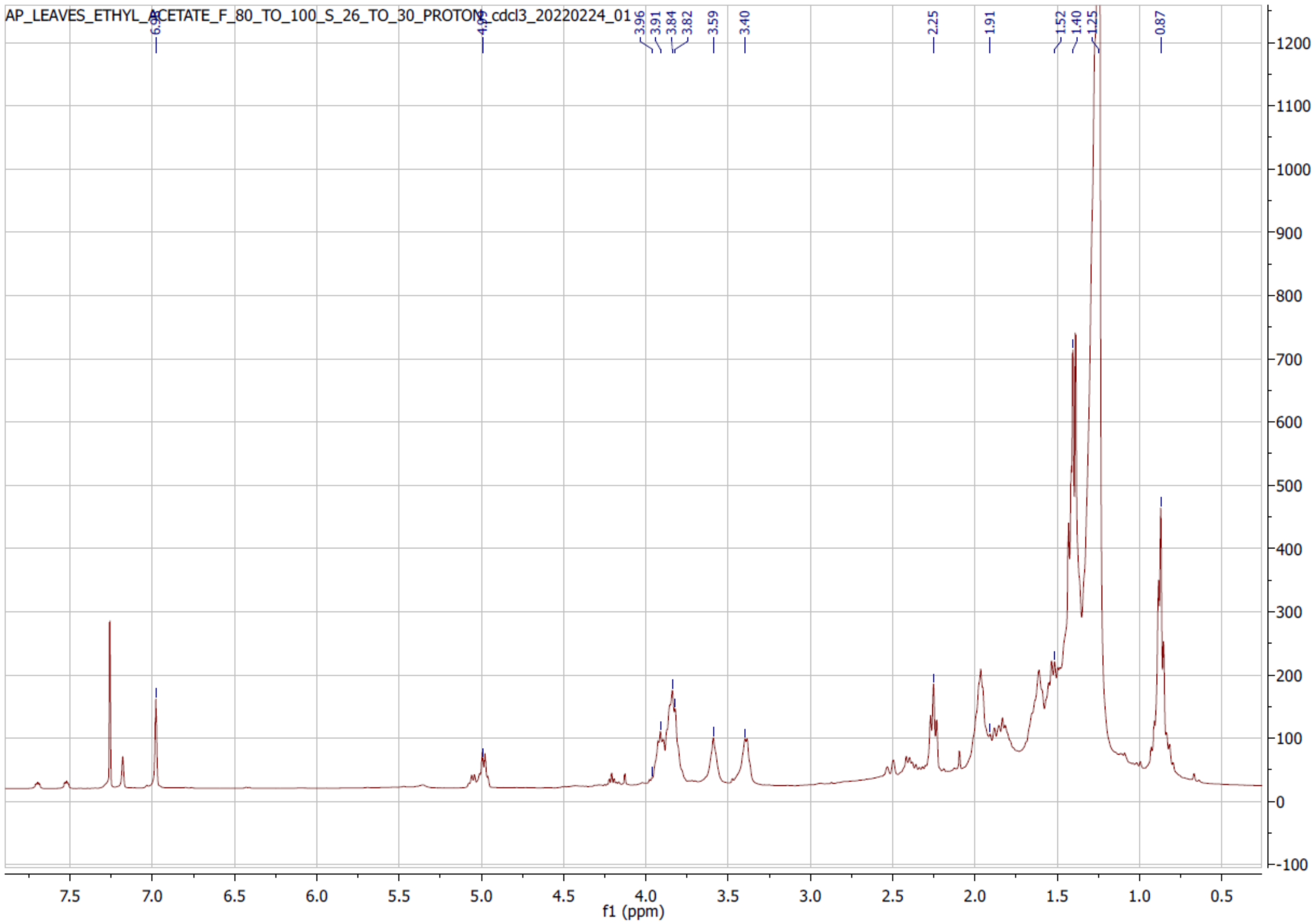

Figure S13: ^{1}H NMR [$CDCl_3$, 400 MHz], chemical shift for Squamocin C

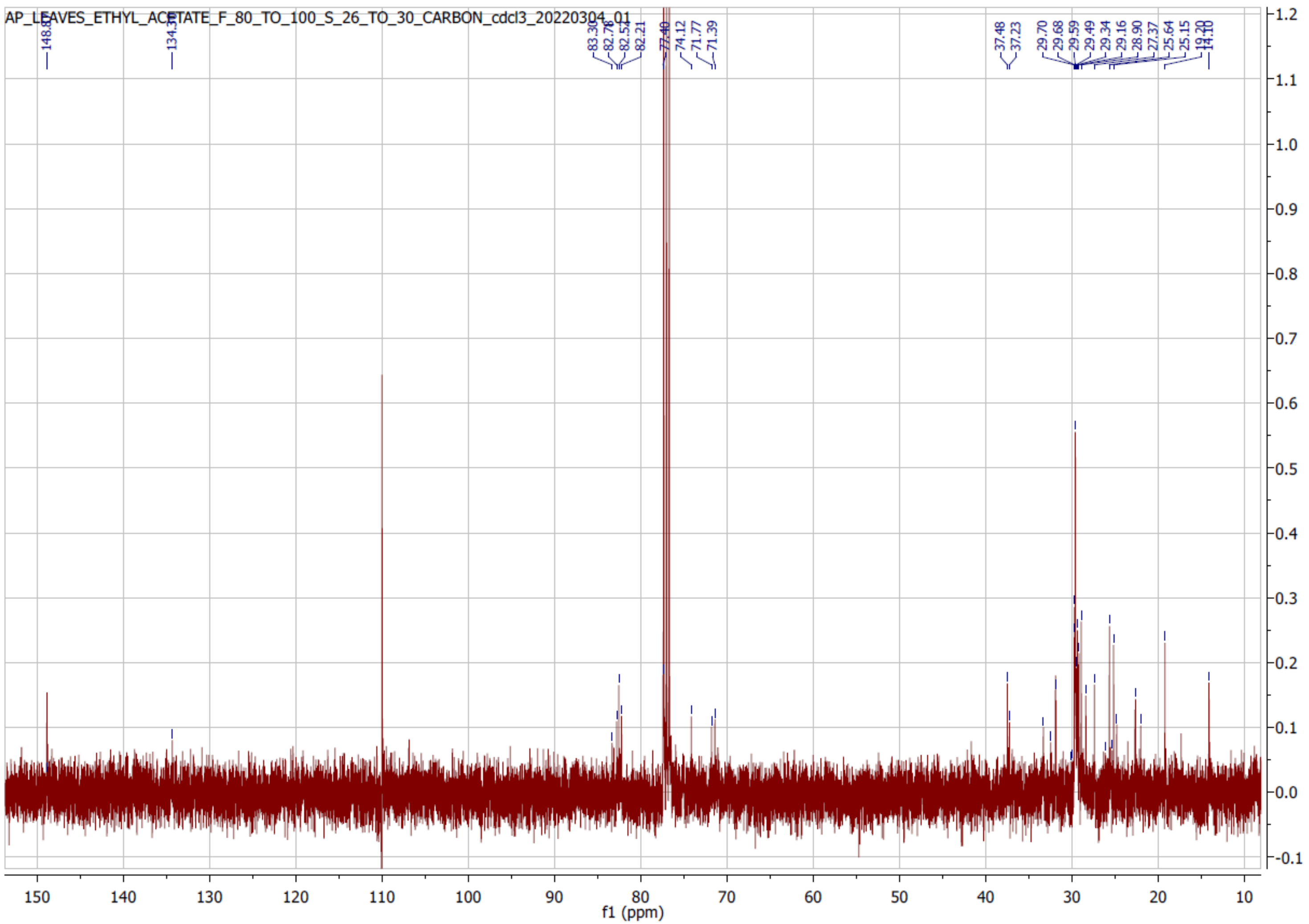

Figure S14: ^{13}C NMR [$CDCl_3$, 400 MHz], chemical shift for Squamocin C

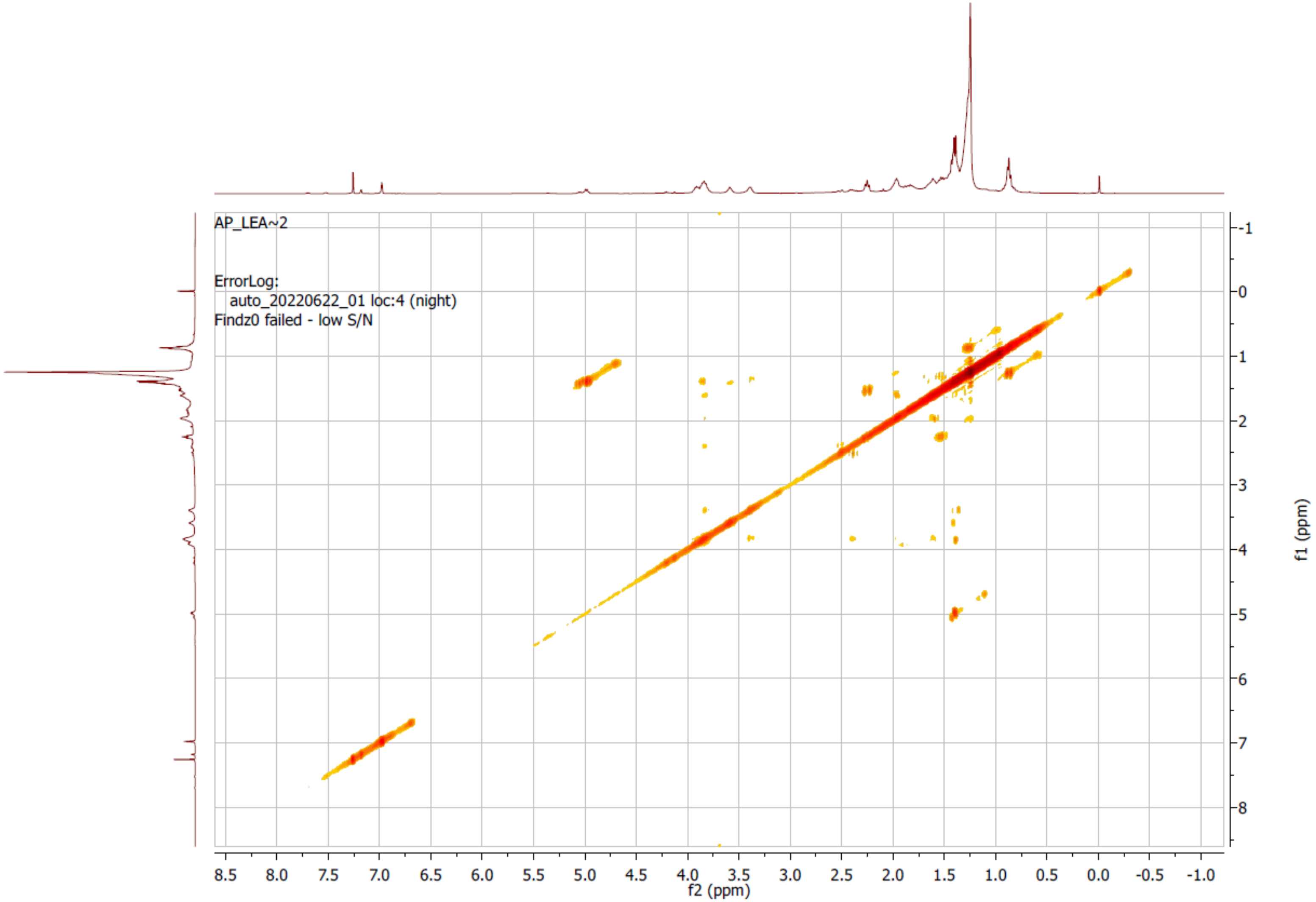

Figure S15: COSY correlation for Squamocin C

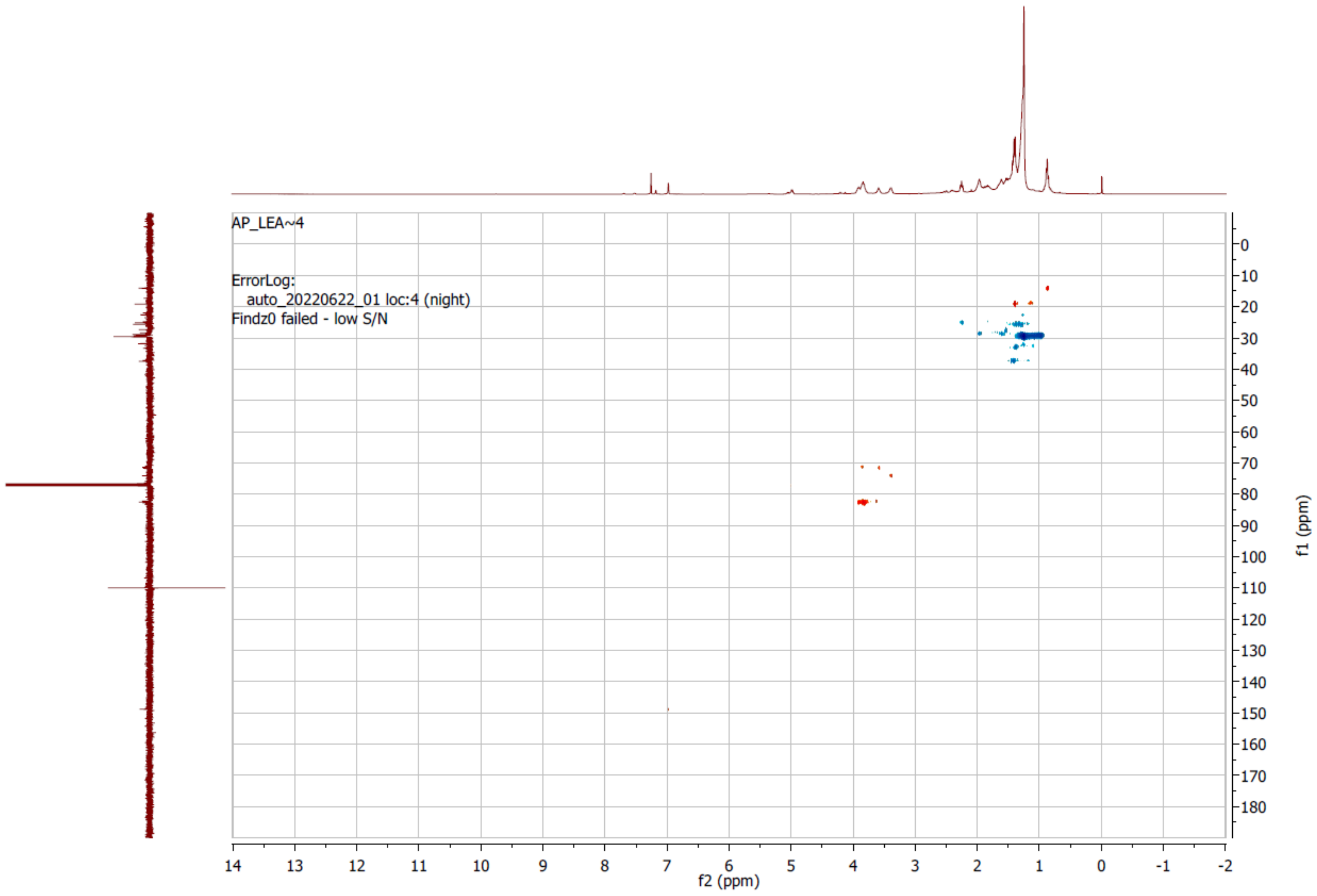

Figure S16: HSQCAD correlation for Squamocin C

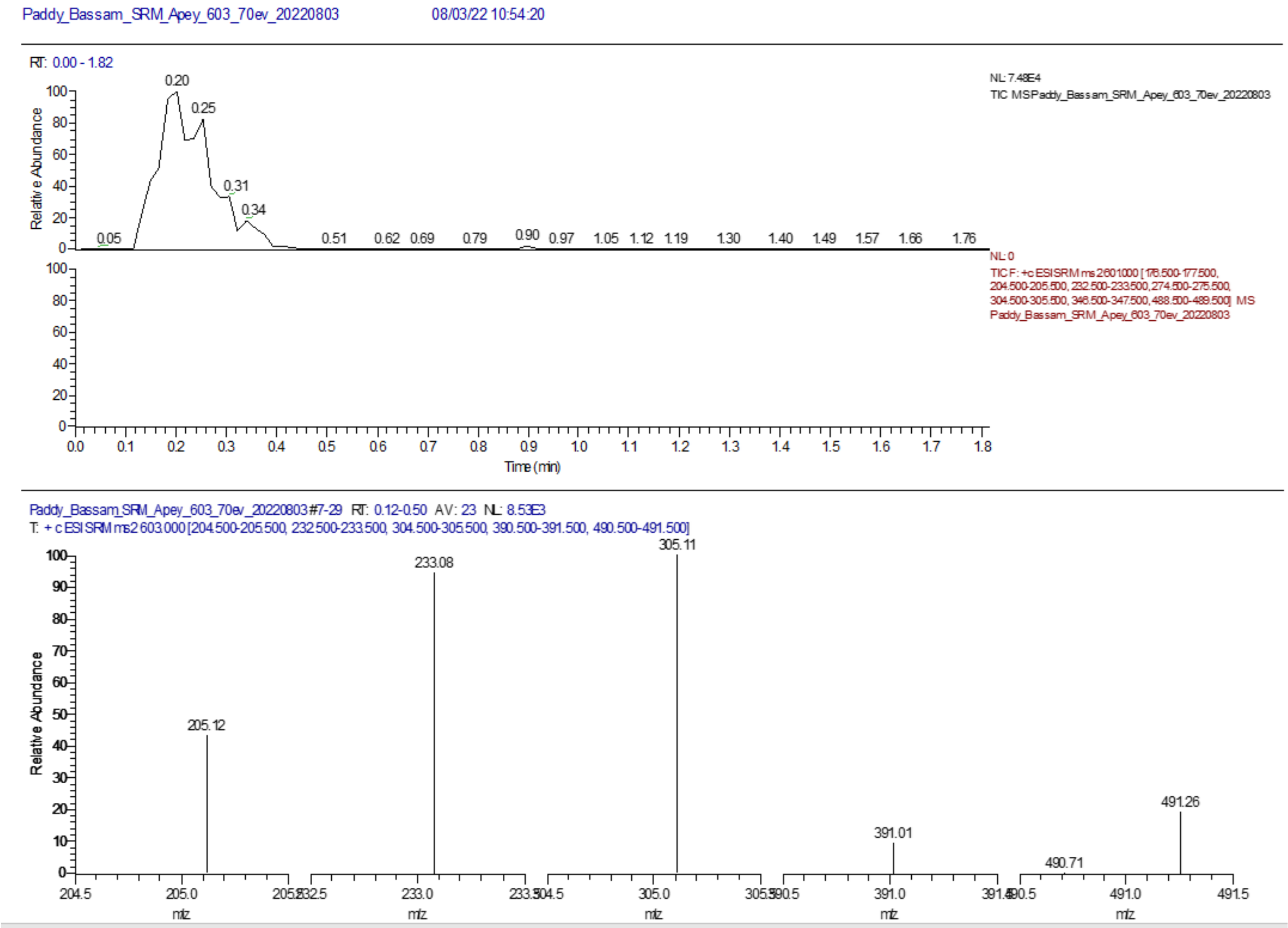

Figure S17: MSMS fragments for Annonacin

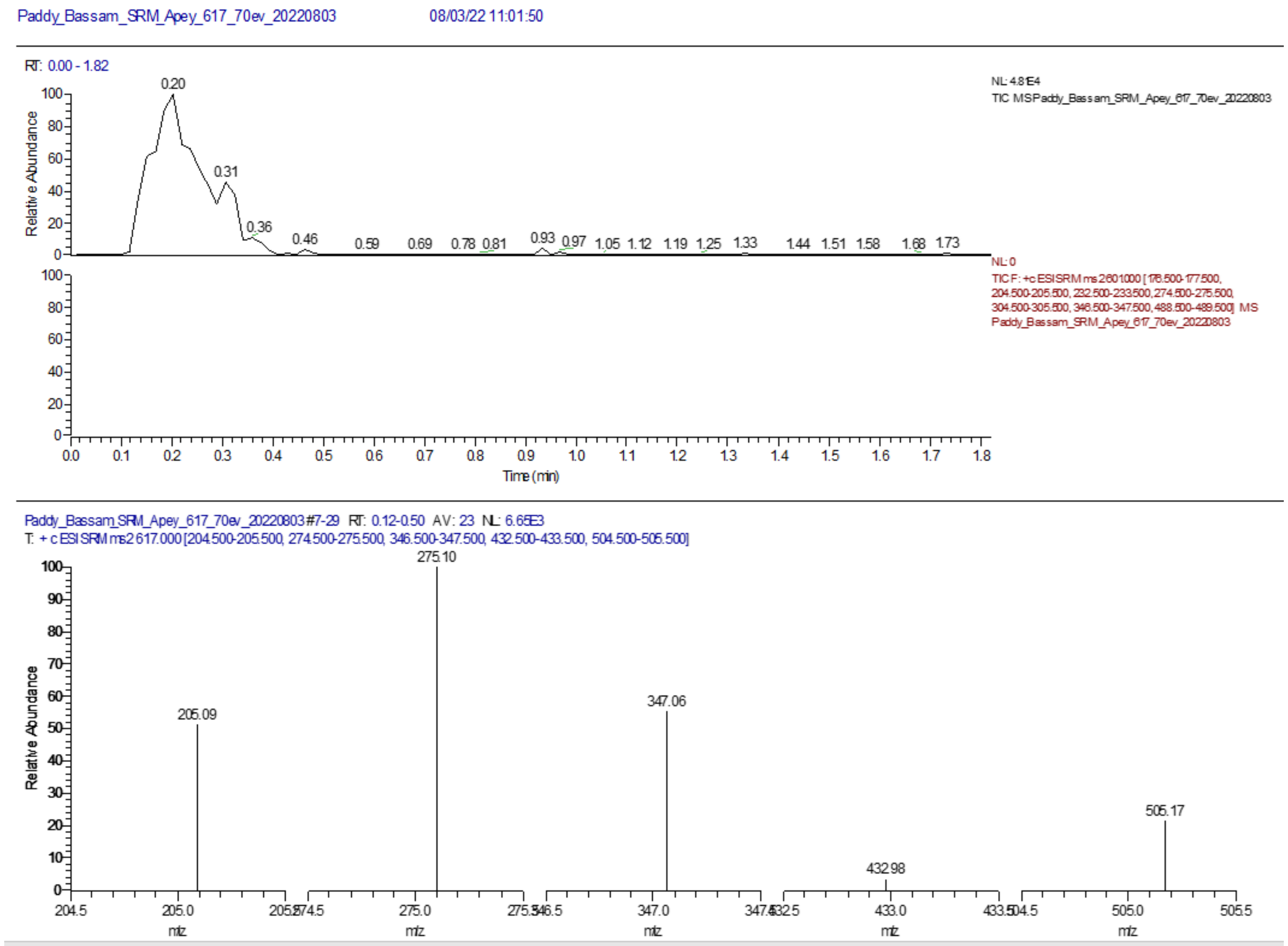

Figure S18: MSMS fragments for Annonisin

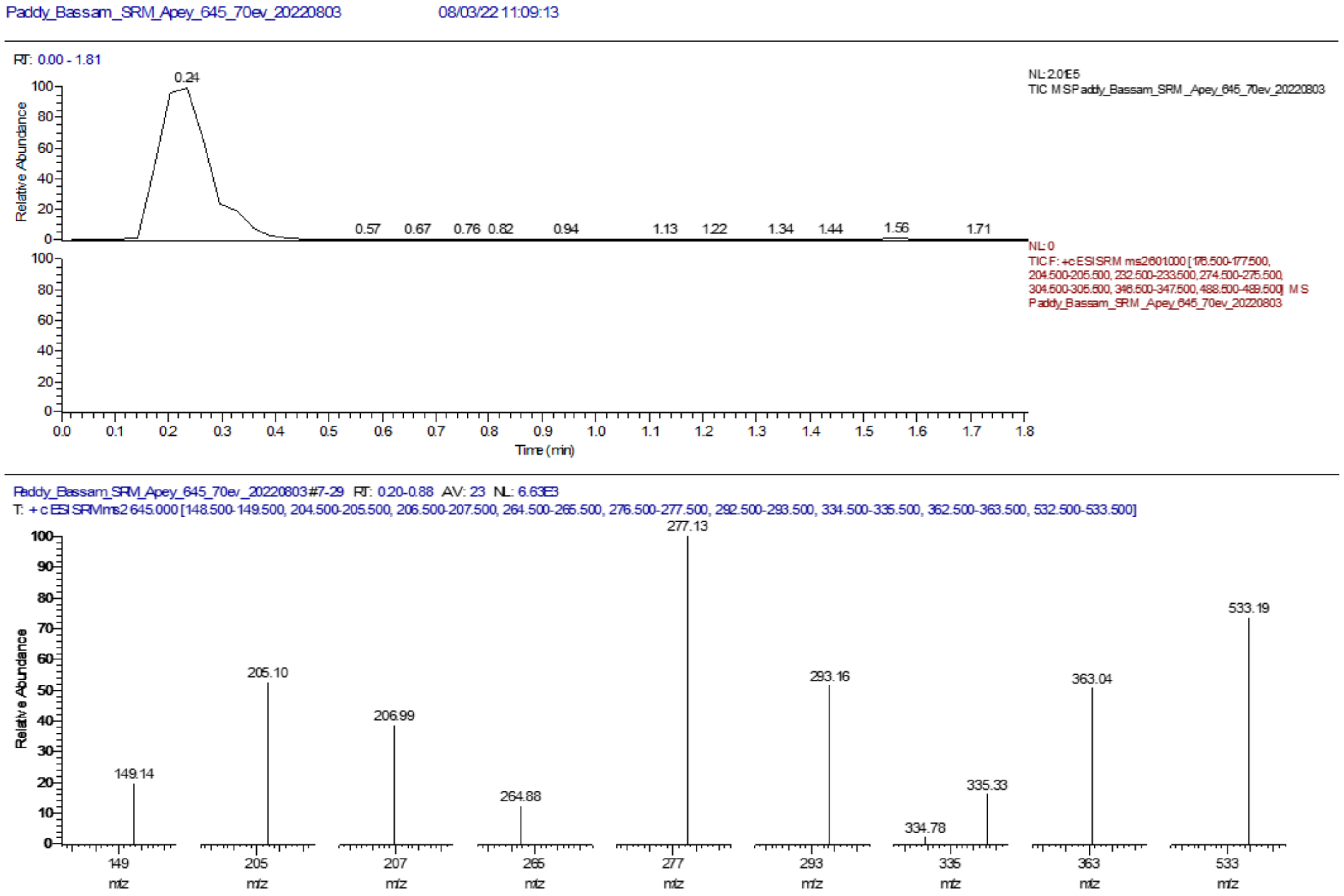

Figure S19: MSMS fragments for Bullatanocin and Atemoyacin-E

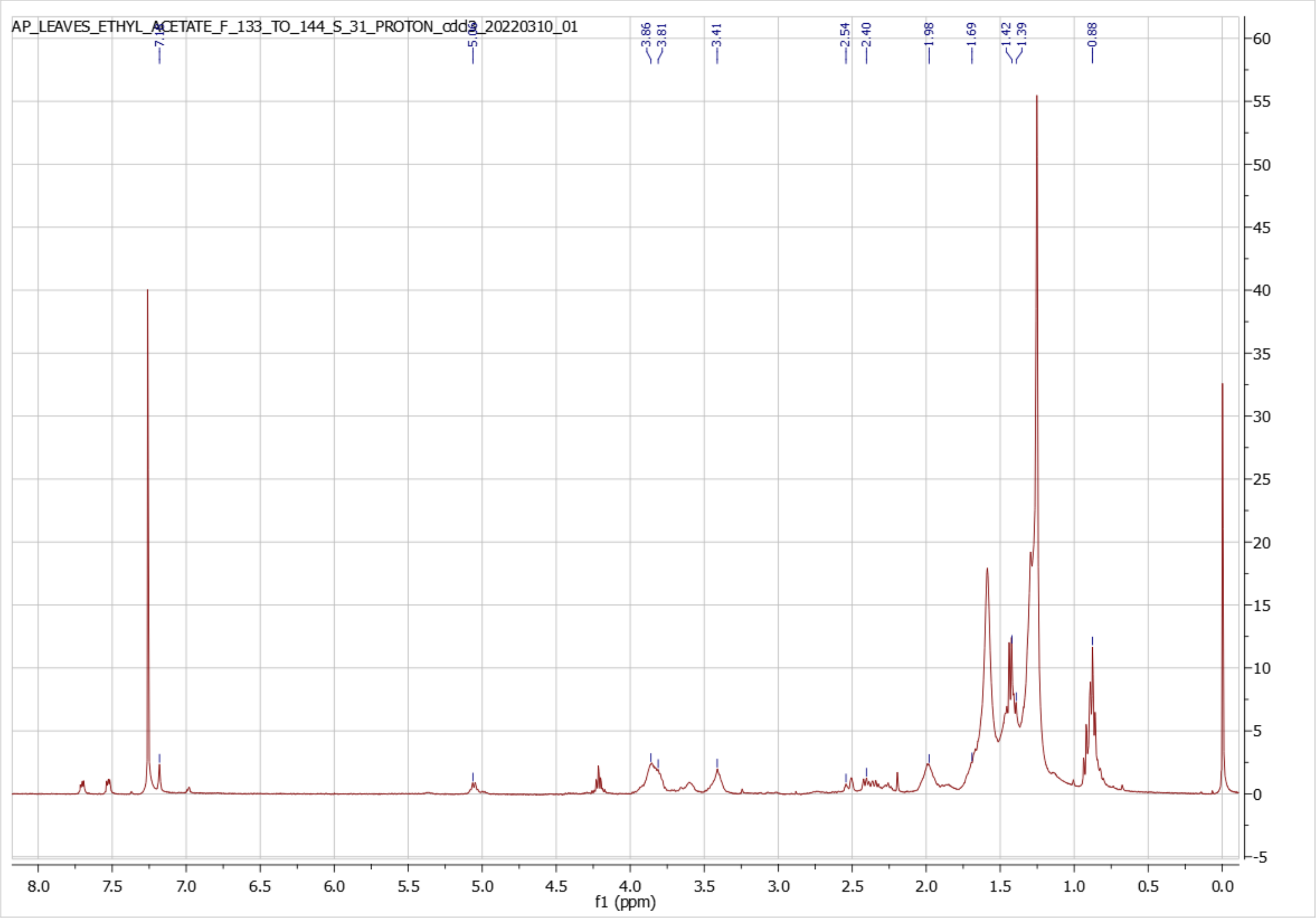

Figure S20: ¹H NMR [$CDCl_3$, 400 MHz], chemical shift for Bullatanocin

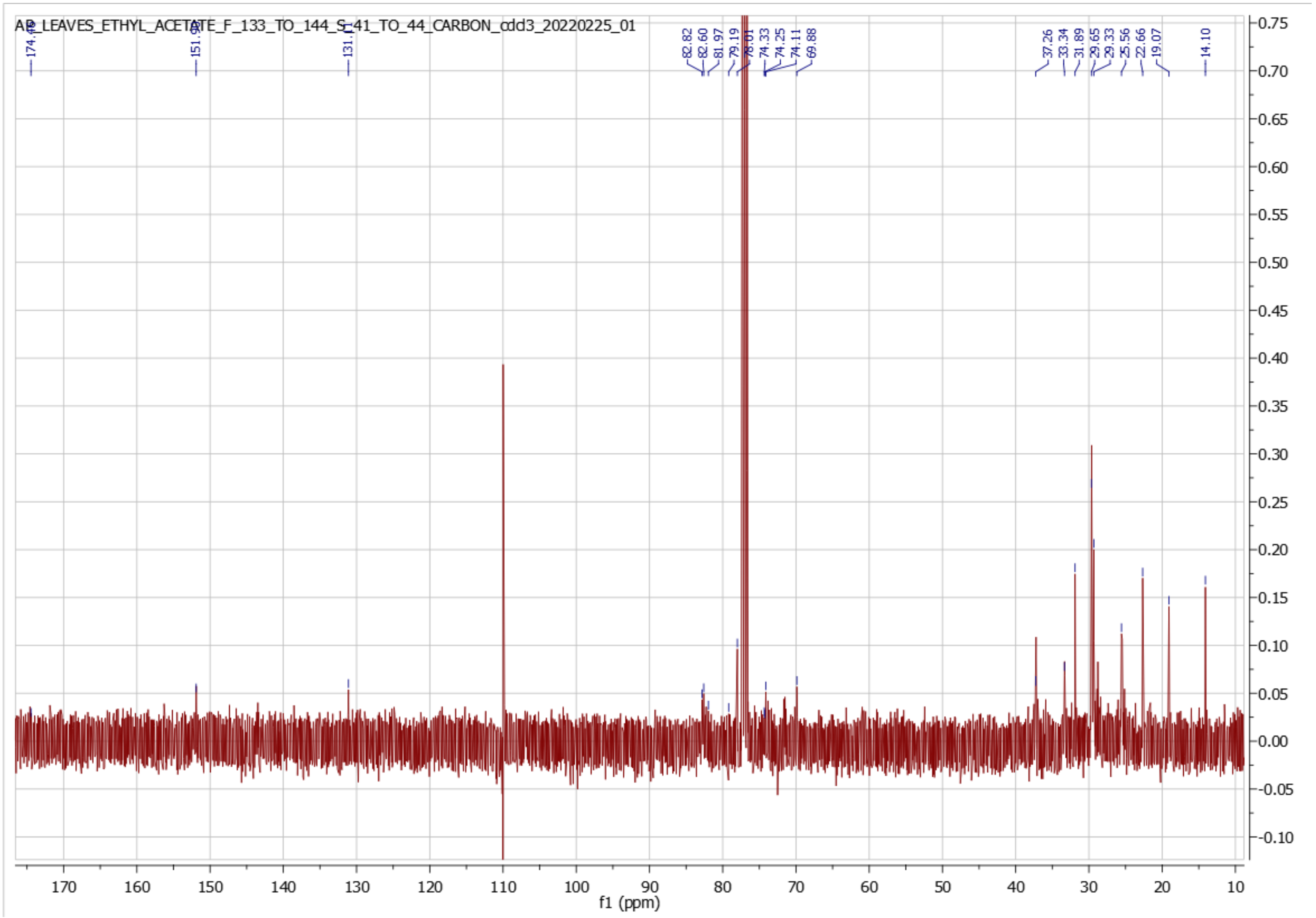

Figure S21: ^{13}C NMR [$CDCl_3$, 400 MHz], chemical shift for Bullatanocin

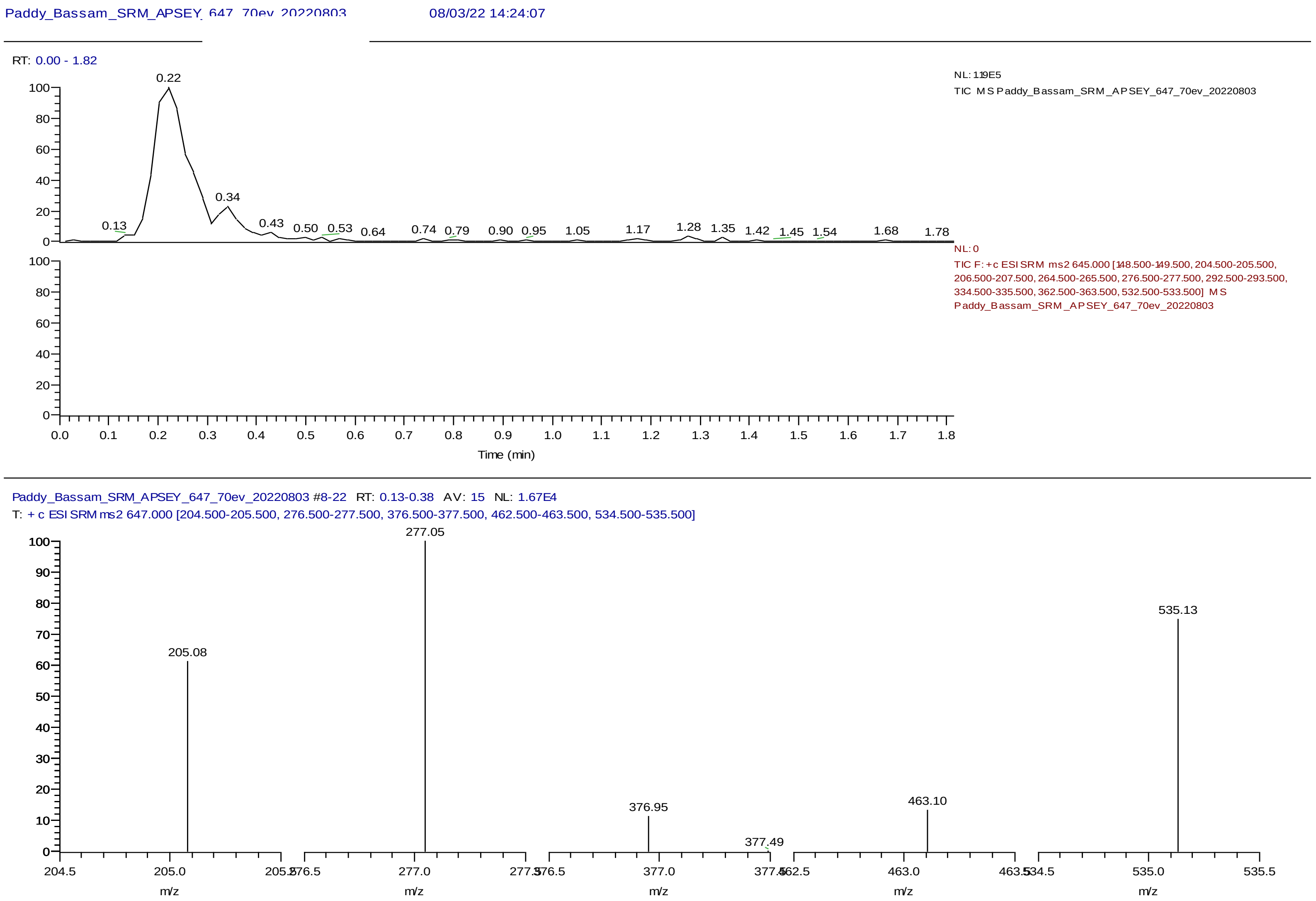

Figure S22: MSMS fragments for Montanacin A

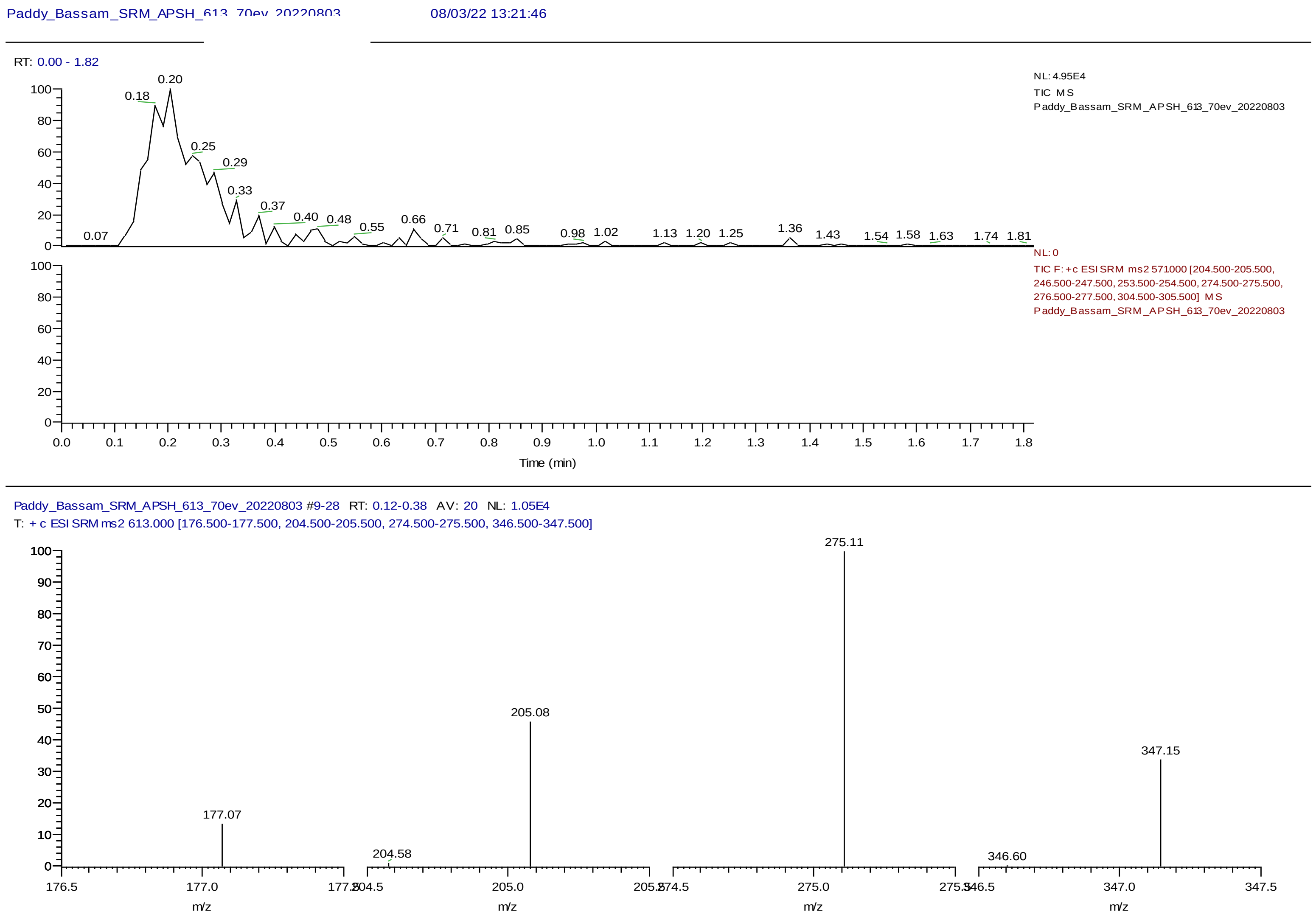

Figure S23: MSMS fragments for Desacetyluvaricin

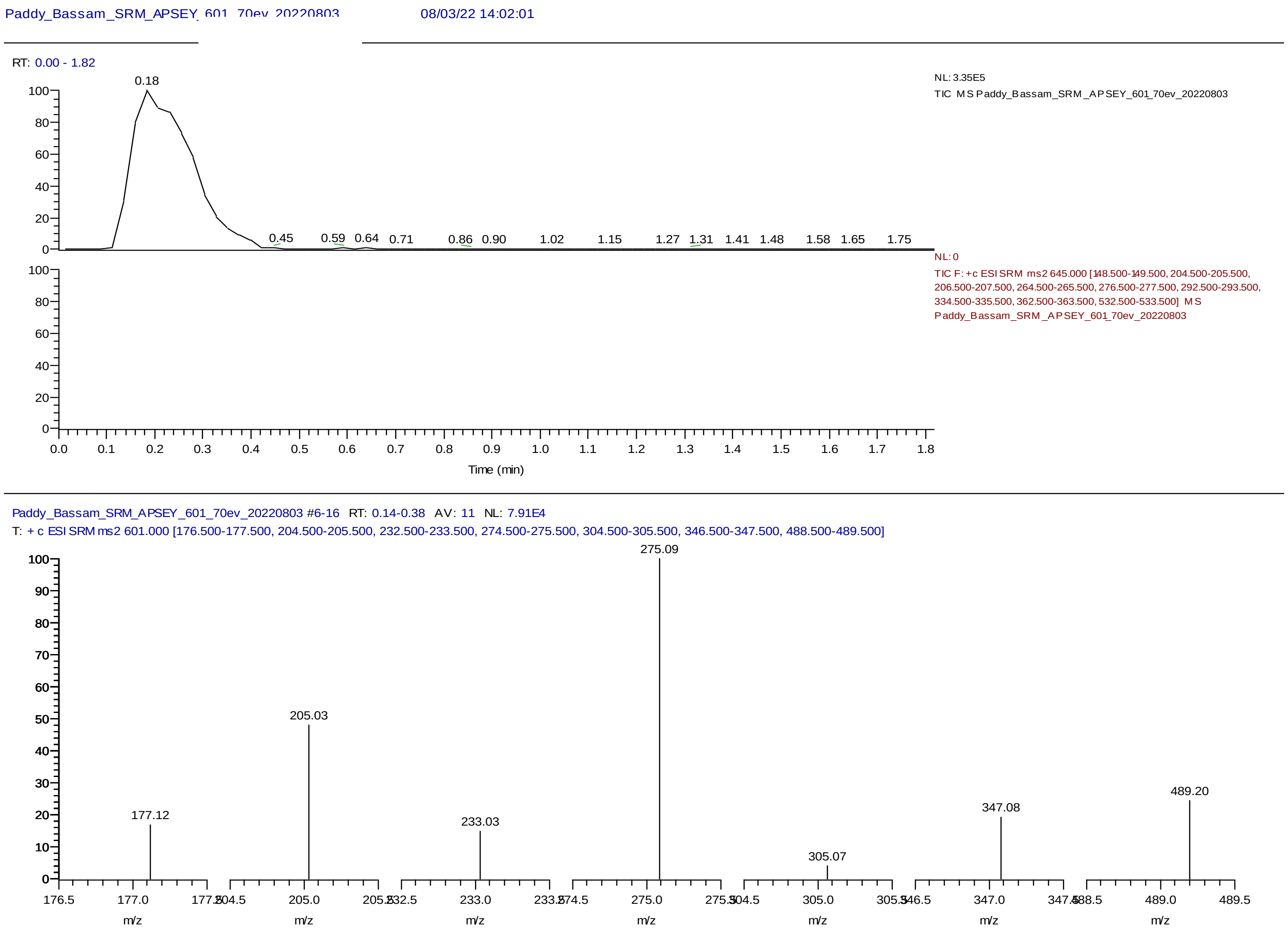

Figure S24: MSMS fragments for Atemoyacin-A

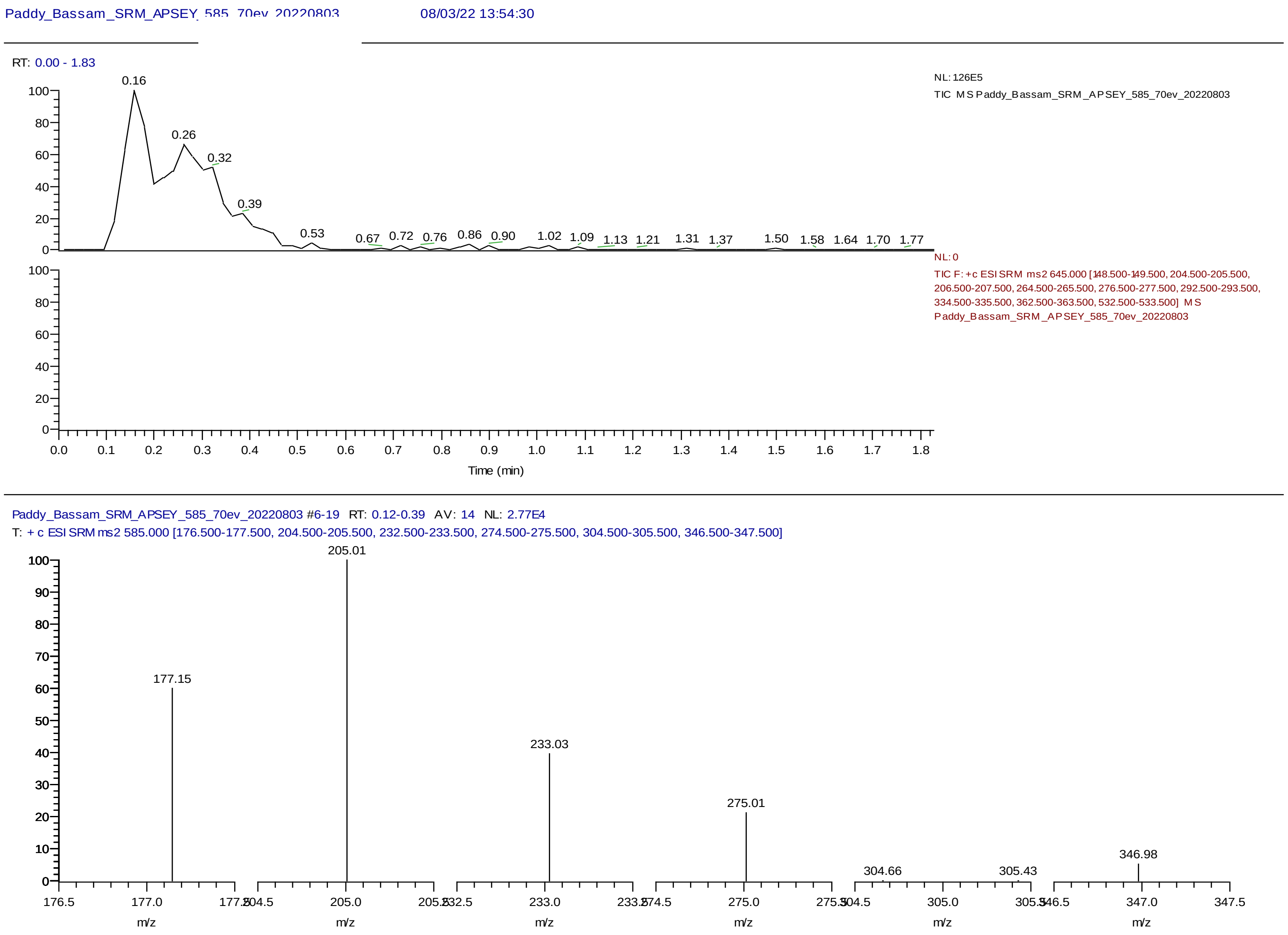

Figure S25: MSMS fragments for Atemoyin

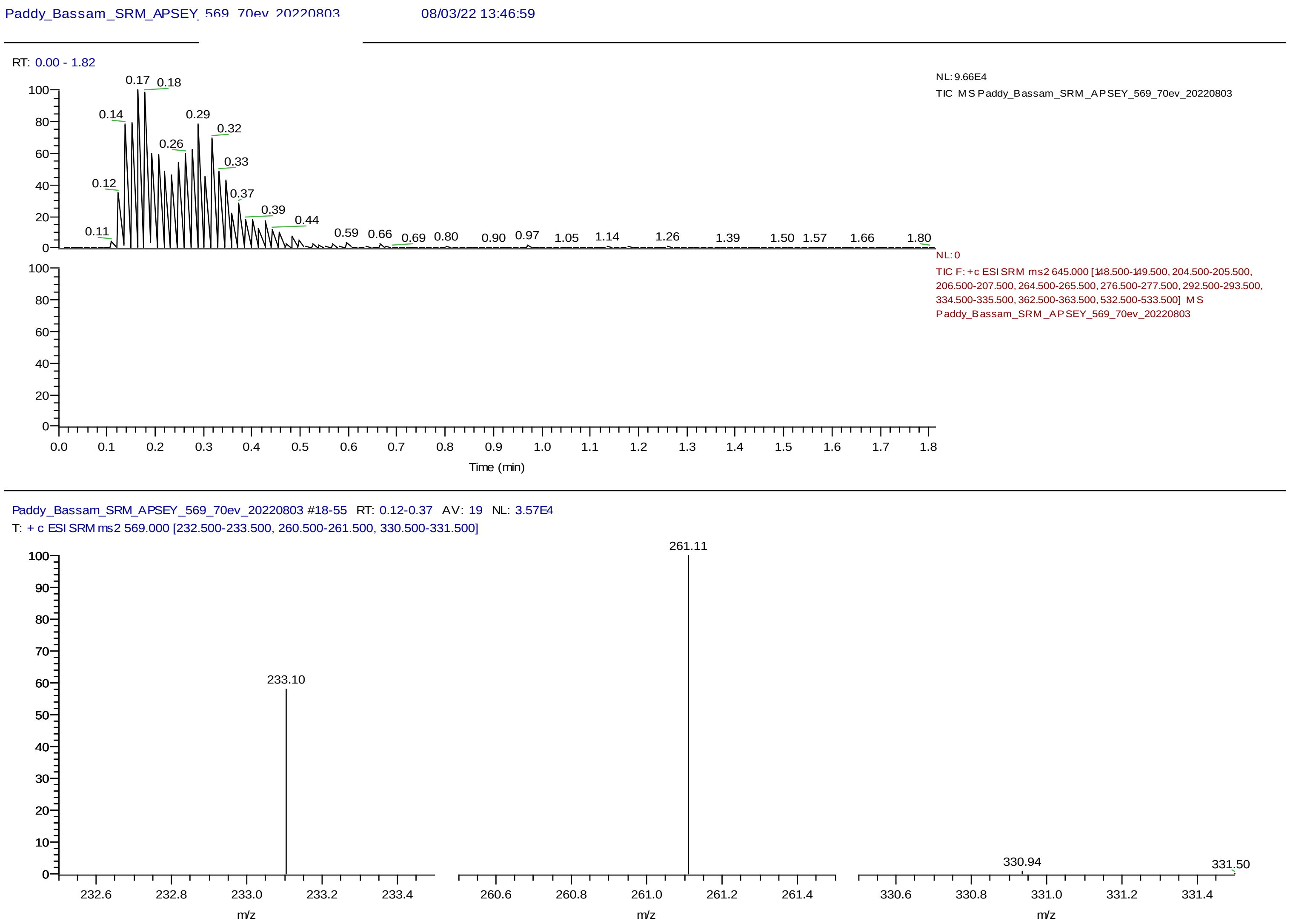

Figure S26: MSMS fragments for Robustocin
